# Using Brain Organoids to Explore Human Neurobiology

**DOI:** 10.1007/s10571-026-01808-5

**Published:** 2026-09-18

**Authors:** Hany E. Marei, Anwarul Hasan

**Affiliations:** 1 Department of Cytology and Histology, Faculty of Veterinary Medicine, Mansoura University, Mansoura, 35116 Egypt; 2 Department of Bioengineering, King Fahd University of Petroleum & Minerals, Dhahran, 31261 Saudi Arabia; 3 Interdisciplinary Research Center for Bio Systems and Machines, King Fahd University of Petroleum & Minerals, Dhahran, 31261 Saudi Arabia

**Keywords:** Brain organoids, Human neurodevelopment, Corticogenesis, Neural stem cells, Pluripotent stem cells, Neurodevelopmental disorders, Cortical progenitors, Outer radial glia, 3D in vitro models, Neural circuit formation, Synaptic maturation, NMDA receptor development, Multi-omic analysis, Chromatin architecture, Transcriptional regulation, Epigenetic maturation, Clonal lineage tracing, Cellular heterogeneity, Developmental trajectories, Increasingly complex neural network activity, Organoid transplantation, In vivo integration, Human–rodent chimeric models, Disease modeling platforms, Functional maturation, Mechanistic neuroscience

## Abstract

One of the major challenges in neuroscience is understanding how the human brain develops into a highly organized and functionally integrated organ, because many developmental processes cannot be investigated directly in humans and are not fully recapitulated in animal models. Brain organoids derived from human pluripotent stem cells have emerged as powerful three-dimensional (3D) experimental models that recapitulate key features of human neurodevelopment, including regional patterning, cellular diversification, and neural circuit assembly. Recent advances in long-term organoid culture, organoid bioengineering, vascularization, assembloid technology, transplantation, multi-omics, and artificial intelligence have substantially expanded the applications of brain organoids to investigate human neurodevelopment, model neurological disorders, support drug discovery, and advance therapeutic development. Integration of single-cell and spatial multi-omics with computational approaches has enabled robust molecular benchmarking, assessment of developmental fidelity, and evaluation of organoid reproducibility, facilitating direct comparison with primary human fetal brain tissue. However, current organoid systems remain limited owing to incomplete cellular and tissue complexity, inter-organoid variability, limited vascularization, limited functional maturation, and incomplete physiological integration. Continued advances in tissue engineering, computational biology, and standardized differentiation protocols are expected to enhance the biological fidelity and translational utility of brain organoids for basic and translational neuroscience, as well as mechanistic and translational research.

**Graphical Abstract:**

**Figure:**
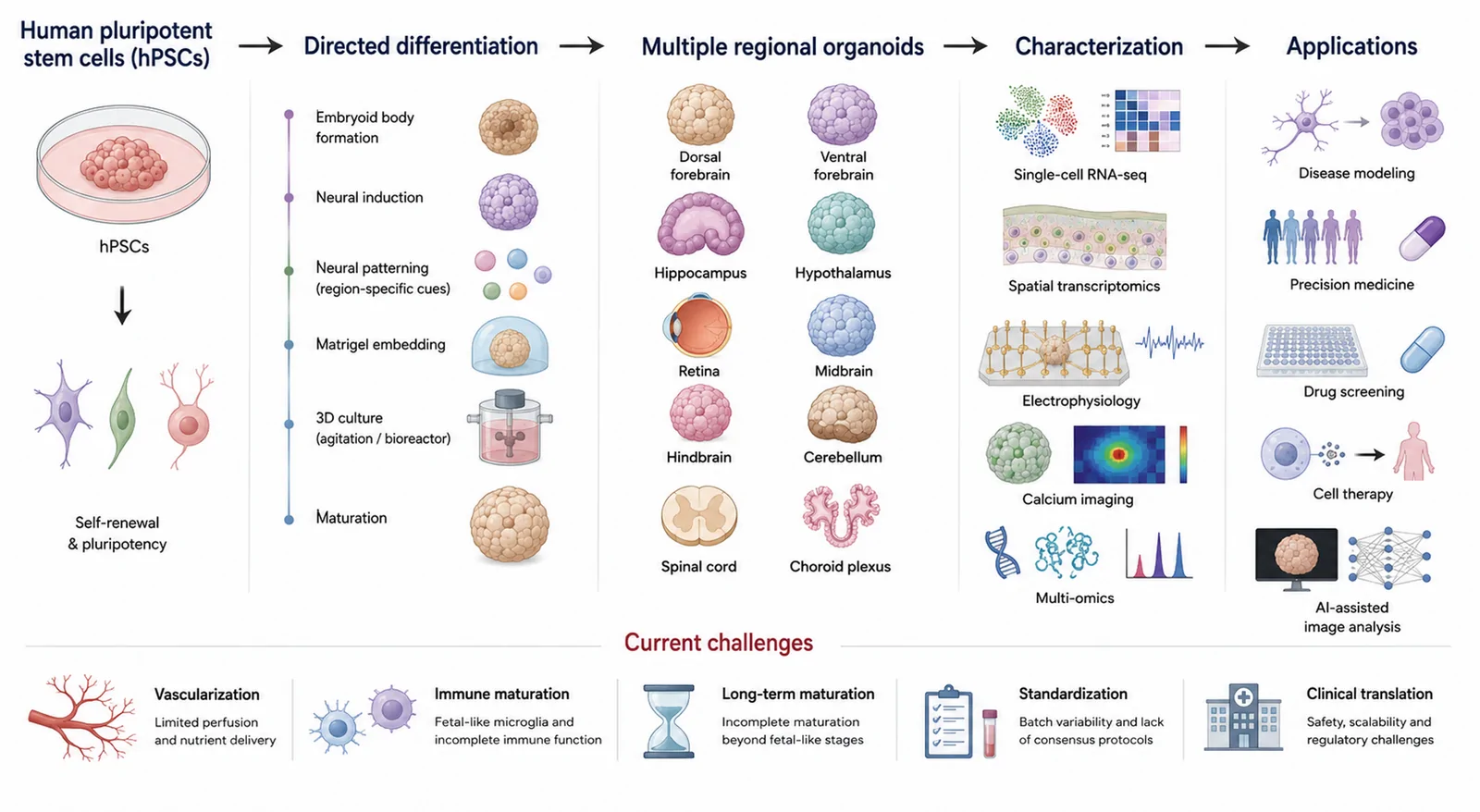

Human Pluripotent Stem Cell-Derived Brain Organoids: From Directed Differentiation to Biomedical Applications. The graphic presented represents a summary of the overall workflow used for creating, characterizing, and medical uses of the brain organoids derived from stem cells. To create brain organoids, scientists utilize stem cells, and through controlling developmental signaling pathway processes bring forth the organoids that are organ-specific and exhibit distinct features of formation of human body. Depending on the technique that was used for creating organoids, different kinds of organoids can be generated, including, for instance, the organoids of the dorsal forebrain (one can also name it as the cortex), organoid of the ventral forebrain, organoids of different parts of the brain like stipular, hypothalamic, retinal, mesencephalic, rhombic, cerebellar, and spinal organoids. There are many methods that help with providing enough information about organoids. The methods used mostly can be characterized by their efficiency, using approaches that enable scientists to know more about cell heterogeneity through using scRNA-seq, understanding the tissue structure through the use of spatial transcriptomics, learning about neuron functioning through utilizing electroactivity tests and calcium imaging, and obtaining other information through applying multiple omics. Organ identity becomes interactive with many branches of science without exception, mostly forming large platform for research and applications in the field of neuroscience. But still, the process of applying brain-based approaches for research development requires the solution of some issues that need to be attended and solved. Most of the issues emerged can be explained by a lack of blood vessels, partial immune system efficiency, limitations in cell functioning, and variability of protocols. Summarizing the graphic provided, it is evident that it represents a detailed process of brain organoid research and its application. The information provided for making the above-mentioned graphic was based on numerous scientific works that deal with the issue of the generation of organoids and the general overview of the development, usage, and creation process of this material. Abbreviations: AI, artificial intelligence; hPSC, human pluripotent stem cell; scRNA-seq, single-cell RNA sequencing.

## Introduction

Understanding human brain development remains one of the major challenges in neuroscience. The development of the human brain requires precise spatiotemporal coordination of neural stem cell proliferation, cell lineage specification, neuronal migration, synapse formation, and neural circuit assembly. Even subtle perturbations in these processes can lead to neurodevelopmental or neuropsychiatric disorders that remain incompletely understood, despite decades of intensive investigation. Animal models, particularly rodents, have provided fundamental insights into neural development, but their ability to recapitulate human brain development is limited by anatomical, cellular, and molecular differences.

The human cerebral cortex differs substantially in size, cytoarchitecture, and cellular complexity from that of rodents. While the mouse brain is lissencephalic, the human cerebral cortex is gyrencephalic and contains substantially more cortical neurons. This expansion is attributed to several human-specific developmental features, such as the abundance of outer radial glial (oRG) cells in the outer subventricular zone and human-specific gene regulatory programs that distinguish primate cortical development (Bystron et al. 2008; Hansen et al. 2010; Chiaradia and Lancaster 2020). Additionally, Ethical and practical constraints limit direct investigation of brain development. These limitations have driven the development of complementary human-specific experimental models.

Brain organoids are three-dimensional (3D) in vitro models derived from human pluripotent stem cells. Through intrinsic self-organization, these brain organoids recapitulate key features of early human neurodevelopment, including regional organization, neuroectodermal architecture, progenitor cell diversity, neuronal differentiation, and neural circuit formation.

These organoids can be experimentally manipulated through genetic, environmental, and biochemical perturbations to study normal human development and the causes of neurological disorders. Organoid technology has advanced rapidly, substantially expanding its experimental utility in scientific fields. The advances in long-term organoid culture, bioengineering, organoid vascularization, methods for organoid transplantation, assembloid technology, and single-cell and spatial multi-omics technologies have substantially expanded the applications of brain organoids in modeling complex processes underlying human brain development and function (Gordon et al. 2021; Noack et al. 2023; Lindenhofer et al. 2024). Nevertheless, such advancements have revealed various issues associated with organoid variability, immaturity, limited vascularization, and the lack of standardized validation frameworks.

**Fig. 1:**
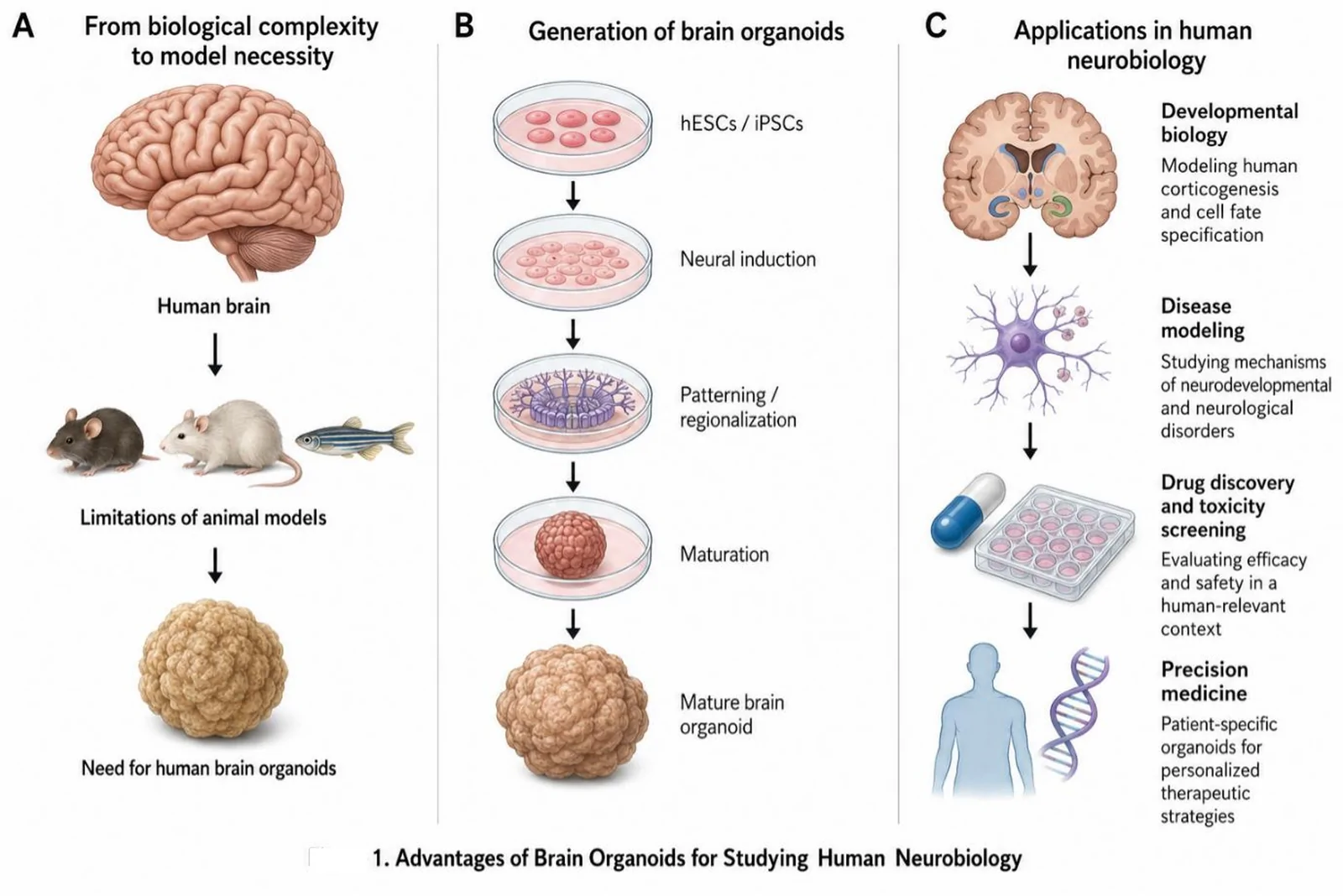
Advantages of Brain Organoids for Studying Human Neurobiology. This figure outlines several major benefits of using brain organoids as experimental models for studying human neurodevelopmental and neurological diseases. Brain organoids can provide a human-specific means of studying brain development in ways that may not be possible with traditional animal models. Brain organoids are derived from human pluripotent stem cells (hPSCs), which can be derived from human embryonic stem cells (hESCs) or induced pluripotent stem cells (iPSCs) via sequential neural induction, regional specification, and maturation. The use of brain organoids has significantly expanded the possibilities for studying various aspects of human neurodevelopment, including disease modeling, drug development, and personalized medicine. The figure emphasizes that the key advantage of organoids is that they allow for the development of reproducible human-specific investigations with results that are more relevant to human neurodevelopment and neurological diseases than those from studies conducted on animals, which enables scientists to investigate the cellular and molecular mechanisms underlying many aspects of human neurodevelopment and neurological diseases. The figure depicts advances in human genetics, functional genomics, stem cell biology, organoids and microphysiological systems, genome editing, single-cell omics, systems biology, and artificial intelligence (Lancaster and Knoblich 2014; Velasco et al. 2019; Gordon et al. 2021; Noack et al. 2023; Zenk et al. 2024)

In this Review, we examine recent advances in brain organoid technologies and their contributions to human neurobiology. We describe how brain organoids are generated and used in research on neurodevelopment and neurological disease (Fig. 1). We also discuss recent advances in assembloid technology, bioengineering, and transplantation, and the use of multi-omics and computational approaches in brain organoid studies, while outlining the biological, technical, computational, and ethical challenges.

## Conceptual Foundations of Brain Organoids

Human brain development proceeds through a precisely coordinated sequence of spatiotemporal events that governs neural stem cell proliferation, cell lineage specification, neuronal migration, and circuit formation. These processes are regulated by dynamic gene regulatory networks that interact with morphogen gradients, mechanical cues, and intercellular signaling to establish regional brain identities and ultimately generate the complex cytoarchitecture of the cortex (Chiaradia and Lancaster 2020). These developmental principles provide the conceptual foundation for brain organoid technology, which aims to recapitulate human brain development.

Neurogenesis begins in the ventricular zone (VZ), where neuroepithelial cells differentiate into radial glial (RG) progenitors, which are the principal neural stem cells of the developing cortex (Kriegstein and Alvarez-Buylla 2009; Chiaradia and Lancaster 2020). In early embryonic development, RG cells primarily divide symmetrically, thereby expanding the progenitor pool. As corticogenesis progresses, RG cells switch from symmetric to asymmetric divisions, producing one self-renewing RG cell and either a neuron or an intermediate progenitor (IP) cell (Rakic 1972; Bystron et al. 2008, pp. 110–122). This regulated transition is critical for establishing the appropriate number and diversity of cortical neurons.

As cortical development continues, proliferative activity progressively shifts to the subventricular zone (SVZ) and, therefore, becomes the dominant germinal site through the middle of pregnancy. The SVZ contains two principal progenitor populations: intermediate progenitors that amplify neuronal production and outer RG (oRG) cells, which exhibit extensive self-renewal capacity and are a defining feature of primate corticogenesis. Unlike rodent brains, oRG cells in the human brain are markedly enriched in the oSVZ, contributing to the evolutionary expansion and increased complexity of the human cerebral cortex and to the generation of upper-layer cortical neurons (Hansen et al. 2010; Chiaradia and Lancaster 2020). Thus, the number of oRGs and their proliferation are considered two of the main factors determining human cortical development and are among the major biological features not reproduced in traditional rodent models.

Newly generated neurons migrate radially on the elongated basal processes of RG cells to the developing cortical plate. Neurons that are formed earliest will populate the preplate and subsequently divide into the marginal zone and subplate. These transient structures subsequently guide neuronal migration and the arrangement of neurons into layers (Rakic 1972; Bystron et al. 2008). Cortical lamination follows an inside-out pattern, meaning deep-layer neurons are generated first, followed by the emergence of newly generated neurons that migrate above older ones, populating the superficial cortical layers. This tightly regulated process ultimately establishes an organized cortex composed of six layers characteristic of the mature mammalian neocortex (Bystron et al. 2008; Chiaradia and Lancaster 2020).

In vivo, however, this series of developmental processes is precisely regulated through coordinated intrinsic and extrinsic regulatory mechanisms, which can be partially recapitulated under appropriate in vitro conditions. Brain organoid technology exploits this intrinsic capacity for self-organization; by using brain organoids, human pluripotent stem cells can be used to activate intrinsic gene regulatory programs to drive regional patterning, progenitor diversification, neuronal maturation, and early tissue morphogenesis (Lancaster and Knoblich 2014; Velasco et al. 2019; Zenk et al. 2024). Thus, it is possible to say that organoids recapitulate many of the properties of corticogenesis, like progenitor zones, radial glial architecture, progressive neurogenesis, and maturation of neurons, thereby enabling investigation of these stages of human brain development that would otherwise remain inaccessible (Gordon et al. 2021; Noack et al. 2023; Lindenhofer et al. 2024).

**Fig. 2:**
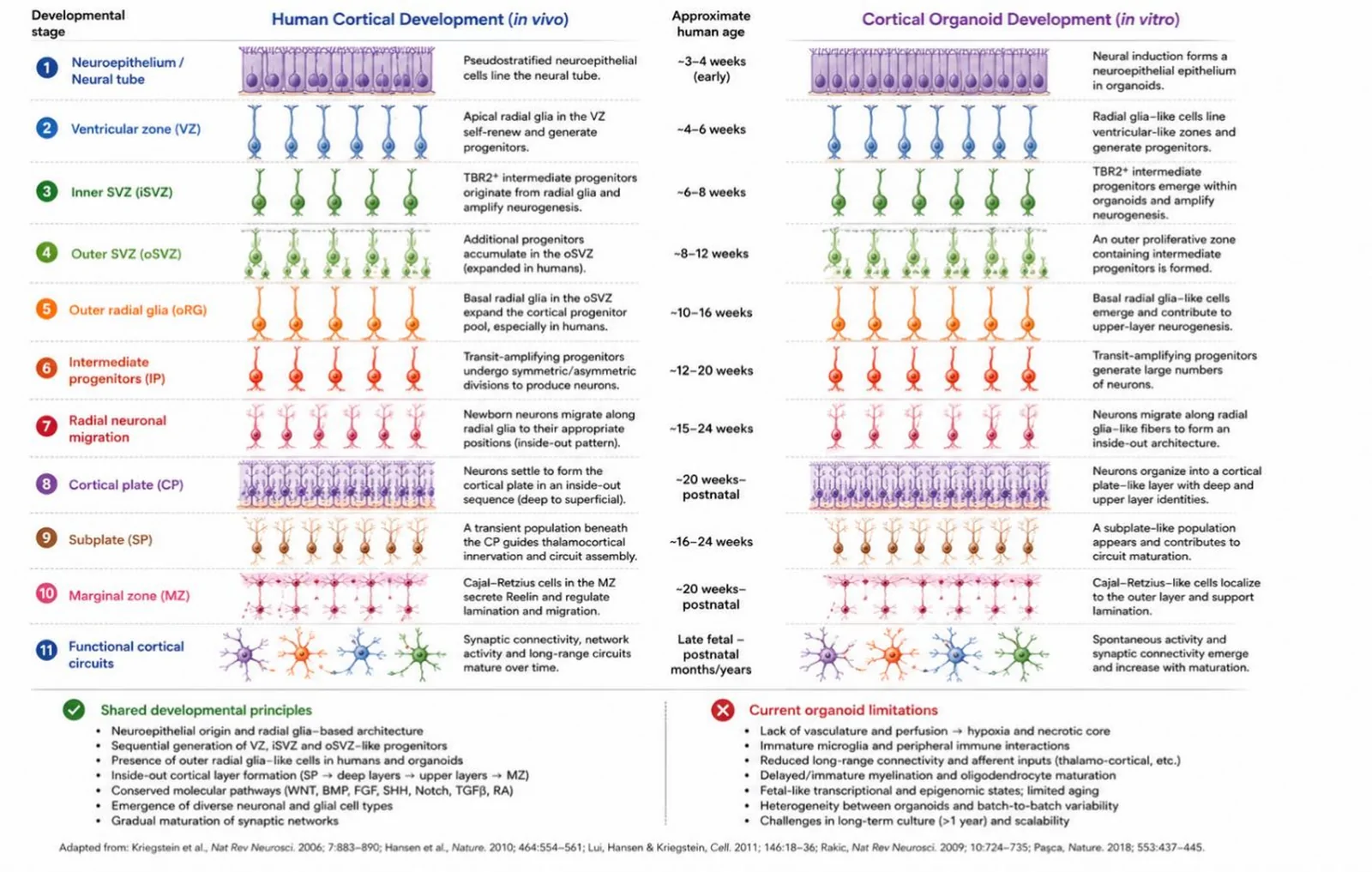
Comparison of Human Cortical Development and Cortical Organoid Development. This figure compares the major developmental stages of the human cerebral cortex in vivo with their corresponding features in human cortical organoids derived from human pluripotent stem cells (in vitro). Parallel developmental timelines illustrate the progression from the neuroepithelium and ventricular progenitor zones through neuronal migration, cortical layer formation, and functional circuit maturation. During corticogenesis, neuroepithelial cells give rise to apical radial glia, intermediate progenitors, and outer radial glia, which sequentially generate excitatory cortical neurons through coordinated proliferation and differentiation within the ventricular zone (VZ), inner subventricular zone (iSVZ), and outer subventricular zone (oSVZ). Newly generated neurons migrate along radial glial fibers to establish the cortical plate (CP), subplate (SP), and marginal zone (MZ), ultimately forming functional cortical circuits. Human cortical organoids recapitulate many of these conserved developmental processes, including progenitor zone organization, neuronal differentiation, radial migration, cortical layer specification, and early neuronal network activity. The lower panel summarizes the major similarities and differences between human cortical development and cortical organoids. Conserved features include neuroepithelial organization, sequential progenitor expansion, emergence of outer radial glia, radial neuronal migration, cortical lamination, and progressive neuronal maturation. However, current organoid models remain limited by incomplete vascularization, immature microglial and glial development, reduced long-range connectivity, fetal-like maturation, inter-organoid variability, and limited long-term physiological maturation. The figure integrates current knowledge of human corticogenesis and cortical organoid development, highlighting conserved developmental mechanisms and the strengths and limitations of organoid models for studying human brain development and disease (Rakic 2009; Kriegstein et al. 2009; Hansen et al. 2010; Lui et al. 2011; Lancaster et al. 2013; Lancaster and Knoblich 2014; Pașca 2015; Chiaradia and Lancaster 2020; Noack et al. 2023; Zenk et al. 2024). Abbreviations: aRG, apical radial glia; CP, cortical plate; IP, intermediate progenitor; iSVZ, inner subventricular zone; MZ, marginal zone; oRG, outer radial glia; oSVZ, outer subventricular zone; SP, subplate; SVZ, subventricular zone; VZ, ventricular zone

While current brain organoids can replicate many essential cellular and molecular features of early corticogenesis, they do not yet fully reproduce the complexity of the human brain in both structure and function. Current limitations of brain organoid methods include incomplete neuronal maturation, limited vascularization, and restricted long-range functional connectivity, while protocol-dependent variability remains substantial (Fig. 2). Still, brain organoids are valuable experimental platforms, as they enable the investigation of multiple aspects of human neurological development (Jourdon et al. 2023; Noack et al. 2023; Zenk et al. 2024).

## Modeling Human-Specific Neurodevelopment

Brain organoids have transformed the study of human neurodevelopment by enabling investigation of developmental processes that are otherwise inaccessible in the human embryo. Compared with traditional animal models, organoids therefore more faithfully recapitulate human morphological processes and enable precise genetic manipulation and the control of environmental and experimental variables under defined conditions. Consequently, researchers now have a powerful experimental platform for studying the general processes involved in human development and investigating human-specific features of human development that cannot be adequately modeled in other experimental systems (Lancaster and Knoblich 2014; Chiaradia and Lancaster 2020).

Early studies of pluripotent stem cell differentiation demonstrated that pluripotent stem cells possess an intrinsic capacity to differentiate into neural lineages under defined culture conditions (Bain et al. 1995; Zhang et al. 2001; Pankratz et al. 2007). Although the embryoid body model and two-dimensional culturing demonstrated neural differentiation, they lacked the three-dimensional architecture required to recapitulate complex neurodevelopmental processes, model regional specification, and support neural circuit formation (Brickman and Serup 2017). The formation of a system for three-dimensional, self-organizing culture based on serum-free cultures using embryoid body-like structures (SFEBq) and organoids based on the extracellular matrix represented a major advance in developmental neuroscience, enabling the investigation of the fundamental principles of early brain morphogenesis and organization to be established (Watanabe et al. 2005; Eiraku et al. 2011; Lancaster and Knoblich 2014).

One of the greatest strengths of brain organoids in scientific research, perhaps, lies in their ability to provide insights into biological processes of human corticogenesis that are not available in rodent models. Brain organoids recapitulate key features of progenitor cell development, including the generation of outer radial glial cells, the sequential generation of neuronal lineages, and prolonged neurogenesis in the human developing cortex (Hansen et al. 2010; Chiaradia and Lancaster 2020). Brain organoids have provided important insights into the mechanisms of cortical expansion, the generation of upper-layer cortical neurons, and the temporal regulation of their development, which have ultimately improved understanding of what differentiates the human cerebral cortex from other mammalian species (Bystron et al. 2008; Chiaradia and Lancaster 2020).

Recent developments in single-cell transcriptomics, epigenomics, and spatial multi-omics have substantially enhanced the biological relevance of brain organoids. Comparative analyses of primary human fetal brain tissue indicate that modern organoid protocols faithfully recapitulate several gene regulatory networks that underlie developmental programs, lineage specification, and transcriptional trajectories in the first half of gestation (Velasco et al. 2019; Gordon et al. 2021). More recently, integrated multi-omics approaches have generated comprehensive molecular atlases that have further refined these comparisons by allowing the reconstruction of several developmental trajectories at the single-cell level and the identification of previously unrecognized progenitor populations. This provides the possibility to quantitatively compare the cells generated in organoids to those present in primary human brain tissue (Noack et al. 2023; Zenk et al. 2024). Collectively, these advances have established brain organoids as a highly reliable model for studying human-specific processes during development.

The use of brain organoids has substantially expanded our understanding of neuronal development processes and neuronal networks by enabling long-term maturation of neuronal populations and synapses that recapitulate prenatal stages of human brain development (Gordon et al. 2021; Jourdon et al. 2023). Consequently, it has become possible to investigate developmental mechanisms that were previously inaccessible, such as those governing neuronal fate, neural circuit assembly, and synaptogenesis in autism spectrum disorder and other neurodevelopmental disorders (Jourdon et al. 2023).

Brain organoids increasingly recapitulate human neurodevelopment but remain subject to important limitations. The current models experience problems with incomplete neuronal maturation, limited glial diversity and maturation, lack of functional vascular and immune components, and limited long-range neural connectivity. Furthermore, protocol-dependent variability remains substantial. To further improve their utility as experimental models, continued advances in bioengineering and differentiation technologies will be essential (Noack et al. 2023; Lindenhofer et al. 2024; Zenk et al. 2024).

In conclusion, brain organoids have transformed the study of human brain development from predominantly descriptive investigations to one that enables mechanistic investigation of its mechanisms. This has enabled the study of the mechanisms of human development, comparison with primary human developmental tissues, and the performance of mechanistic studies that were previously impossible.

## Integrating Complexity: Beyond Early Development

Although early brain organoids primarily model early stages of human neurodevelopment, advances in culture systems and bioengineering technologies have enabled the generation of organoids with substantially greater structural and functional complexity. Extended long-term culture has been a major advance, as it enables the generation of organoids that progress beyond early neurogenesis and exhibit increasingly complex cytoarchitecture, mature neuronal populations exhibiting spontaneous electrophysiological activity, and functional neural networks, resembling those observed during fetal and early postnatal brain development (Lancaster and Knoblich 2014; Velasco et al. 2019; Gordon et al. 2021).

### Cellular Diversification and Functional Maturation

One of the major advances in modern research on brain organoids involves the progressive expansion of cellular heterogeneity and maturation during long-term organoid culture. Extended organoid culture promotes the emergence of increasingly diverse cellular populations of excitatory neurons, inhibitory interneurons, astroglia, oligodendrocyte progenitors, and sometimes, microglia-like cells (Gordon et al. 2021; Jourdon et al. 2023; Lindenhofer et al. 2024).

Single-cell transcriptomics and multimodal spatial omics studies have demonstrated that both guided and unguided differentiation protocols recapitulate numerous neuronal and glial populations developing during human corticogenesis while continuing to exhibit limitations in late neuronal maturation, glial diversification, and neurovascular development (Noack et al. 2023; Zenk et al. 2024). Additionally, molecular benchmarking has substantially improved quantitative assessment of developmental fidelity and protocol optimization.

**Fig. 3:**
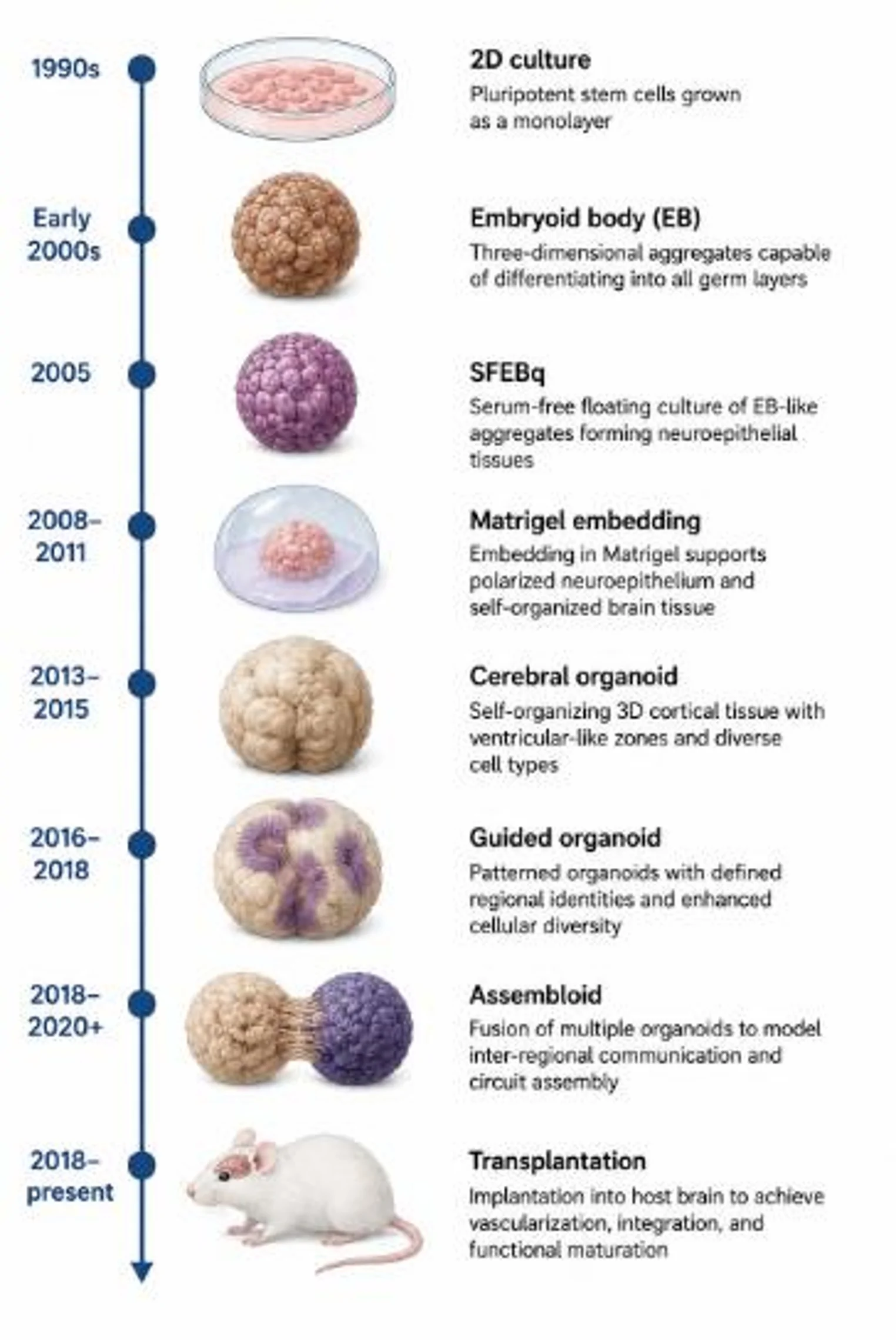
Evolution of Human Brain Organoid Technology. The figure represents the evolution of brain organoid technologies from two-dimensional approaches to three-dimensional methods, including embryoid bodies (EBs), serum-free floating culture of embryoid body-like aggregates with fast reaggregation (SFEBq), cerebral organoids, guided organoids, assembloids, and transplantation models. This evolution has led to greater developmental fidelity, including cellular diversity, regional specification, and functional complexity, thereby achieving a more faithful representation of human neurodevelopment and neurological diseases. The figure illustrates how the development of organoids technologies has expanded the capabilities of human in vitro systems. The conceptual diagram is produced by reviewing and applying state-of-the-art technologies in human genetics, functional genomics, stem cell biology, organoids and microphysiological systems, genome-editing technologies, single-cell and spatial “omics”, systems biology, and AI (Watanabe et al. 2005; Eiraku et al. 2011; Lancaster and Knoblich 2014; Birey et al. 2017; Miura et al. 2022; Reumann et al. 2023)

This increase in cellular complexity is accompanied by progressive functional maturation. The use of long-term cultures promotes dendritic arborization, formation of synapses, spontaneous firing of neurons, generation of oscillatory activity, propagation of calcium waves in addition to coordinated network activity, thus providing evidence for gradual establishment of functional connectivity (Gordon et al. 2021; Jourdon et al. 2023). This has made it possible to investigate neuronal signaling and certain aspects of neural network physiology that could not previously be examined using traditional two-dimensional cultures (Fig. 3).

### Engineering Increasingly Physiological Brain Organoids

Recently, advances in bioengineering have made the brain organoid model increasingly physiologically relevant. Promoting organoid maturation has become more successful with the advanced culture methods used today, such as spinning bioreactors, orbital shakers, air-liquid interface systems, and optimized oxygenation strategies, reducing central necrosis and improving neuronal survival (Gordon et al. 2021; Jourdon et al. 2023).

A significant advancement has been the incorporation of additional cellular components that are underrepresented in conventional organoids in the common organoids. During prolonged culture, astrocytes become increasingly abundant and are of great value in synaptogenesis, neuronal homeostasis, and synaptic pruning. Oligodendrocyte progenitors contribute to axonal myelination under the proper conditions (Kriegstein and Alvarez-Buylla 2009; Gordon et al. 2021; Lindenhofer et al. 2024). The absence of resident microglia in most organoid systems remains a major limitation for research investigating neurodevelopment, including neuroimmune processes (Lancaster and Knoblich 2014; Velasco et al. 2019).

**Fig. 4:**
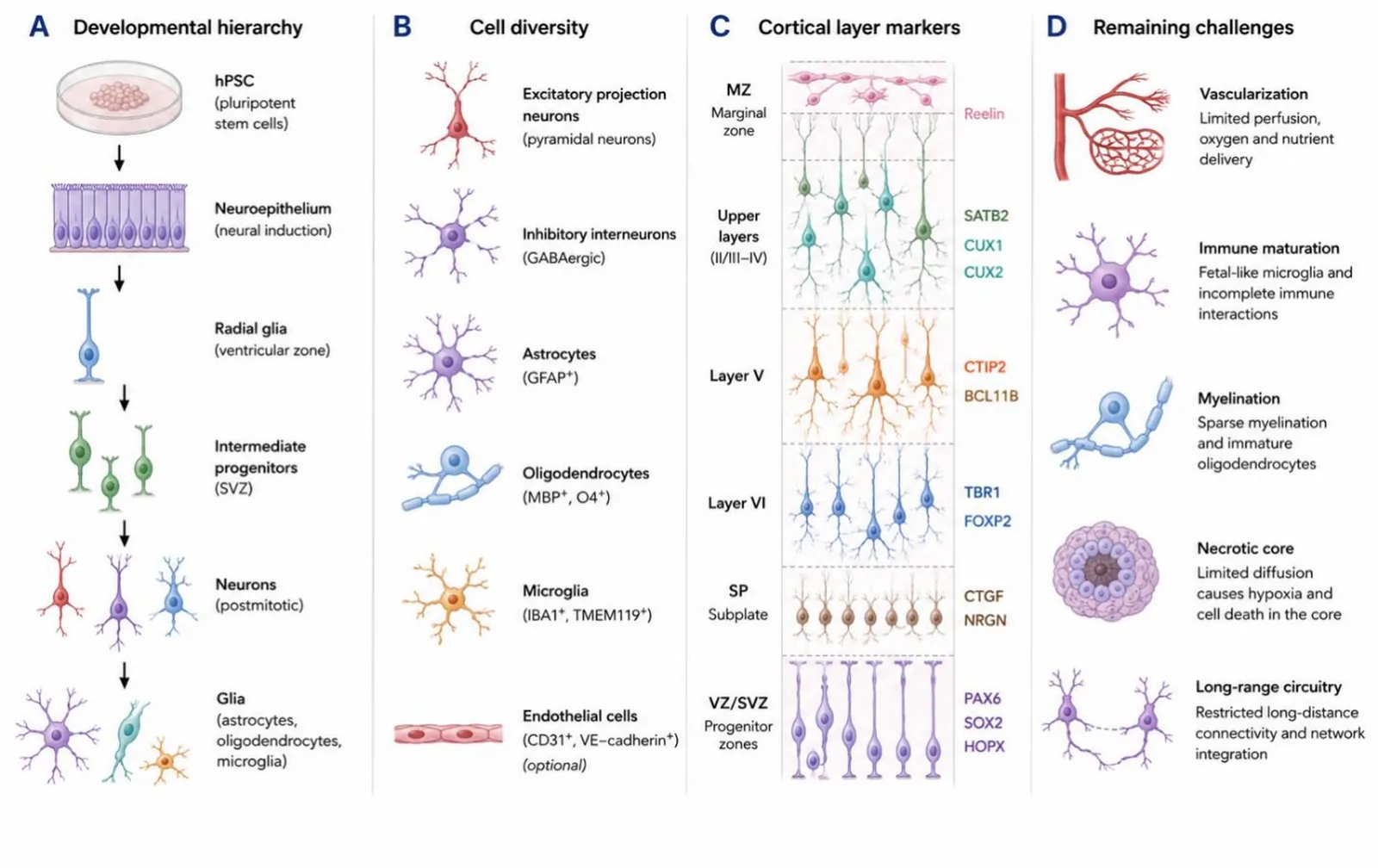
Cellular Organization and Maturation of Human Cortical Organoids. This diagram outlines the processes of human cortical organoids’ development, their cellular diversity, their formation of different cortical layers, and the constraints that exist in the present moment. This form of cells, known as human pluripotent stem cells (hPSCs), undergoes a process of differentiation into neuroepithelial cells, radial glia, intermediate progenitor cells, and cortical glial cells, which are the key stages of human corticogenesis.Aspect **A** of the diagram indicates how human pluripotent stem cells change during the process of maturation. Aspect **B** presents mature cortical organoids that include not only excitatory neurons but also glial cells, which can be both astrocytes, oligodendrocytes, and others. Aspect **C** describes generation of cortical layers where neurons produce TBR1, CTIP2, SATB2, CUX1 neurons in deep and upper layers. Aspect **D** indicates that even though there have been many advancements made in this field at present time, there are still challenges surrounding mature cortical organoids, including lack of blood supply, delayed glial cells’ maturity, limited amounts of immune cells, core necrosis that takes place over time, and decreased capacity for connecting distant neurons. The conceptual model indicated has applied decades of knowledge acquired while working in the field involving the development of human cortex, stem cell differentiation technologies and others (Lancaster and Knoblich 2014; Pașca 2015; Velasco et al. 2019; Gordon et al. 2021; Chiaradia and Lancaster 2020; Jourdon et al. 2023; Noack et al. 2023; Zenk et al. 2024). Abbreviations: BCL11B – B cell lymphoma 11B (CTIP2) CTIP2 – COUP transcription factor-interacting protein 2CUX1 – Cut-like homeobox factor 1CUX2 – Cut-like homeobox factor 2GABA – gamma-aminobutyric acidGFAP – glial fibrillary acidic protein hPSC – human pluripotent stem cellMBP – myelin basic proteinSATB2 – special AT-rich sequence binding proteinTBR1 – T-box brain transcription factor 1

Bioengineering methods have begun to address one of the key biological barriers in organoid systems: the absence of a functional vascular network. Techniques such as endothelial cell co-culture, microfluidic perfusion, organoid-on-chip systems (Zhao et al. 2024; Zhang et al. 2025), and genetic induction of endothelial differentiation have demonstrated the ability to promote partial vascularization and enhance tissue viability (Takebe et al. 2015; Van Duinen et al. 2015; Cakir et al. 2019; Chen et al. 2025; Song et al. 2026). More recently, vascular organoids generated from pluripotent stem cells capable of forming capillary networks have created new opportunities to develop more physiologically relevant neurovascular models (Wimmer et al. 2019). In general, these developments are expected to improve tissue maturation, nutrient delivery, and experimental reproducibility, and enable more efficient disease modeling and drug testing (Fig. 4).

### RemaiBiological Limitationsning 

Despite substantial advances in the development of brain organoids, current organoid systems do not yet fully recapitulate the human brain. While long-term organoid cultures exhibit greater cellular complexity and function, they still show signs of immaturity (i.e., incomplete neuronal maturation, limited vascularization, incomplete myelination, limited neuroimmune complexity, and limited long-range neural connectivity). In addition, protocol-dependent differences as well as types of stem cells contribute to inter-laboratory variability between laboratories, thereby limiting cross-study comparisons (Noack et al. 2023; Zenk et al. 2024).

These limitations are particularly important when interpreting experimental results. Modern organoid techniques recapitulate many features of the early processes of human brain development. However, they do not fully reproduce the structural and functional complexity of the adult human brain. These techniques may serve as experimentally tractable models for testing specific processes underlying development and disease mechanisms, but they must still be validated against primary human tissue samples and other methods (Table 1).

**Table 1:** Overview of terms in Organoid Models: Characteristics, Applications, Advantages, and Limitations

Organoid Type	Brain Region	Major Applications	Strengths	Current Limitations	Representative References
Cerebral organoid	Forebrain	Corticogenesis, neurodevelopment	Self-organization	Limited maturation, no vasculature	(Lancaster and Knoblich 2014; Velasco et al. 2019)
Cortical organoid	Cerebral cortex	Autism, microcephaly	Reproducible cortical identity	Limited inter-regional connectivity	(Lancaster et al. 2013; Velasco et al. 2019)
Midbrain organoid	Midbrain	Parkinson’s disease	Dopaminergic neurons	Ageing not fully recapitulated	(Jo et al. 2016; Fiorenzano et al. 2021)
Hippocampal organoid	Hippocampus	Memory-related disorders	Regional specification	Incomplete circuitry	(Sakaguchi et al. 2015; Miura et al. 2022)
Assembloid	Multiple regions	Migration, circuit assembly	Inter-regional interactions	Fusion variability	(Birey et al. 2017; Miura et al. 2022)
Vascularized organoid	Multiple regions	Maturation, drug testing	Improved viability	Technically demanding	(Mansour et al. 2018; Cakir et al. 2019)

### Future Perspectives

It is anticipated that continued advances in bioengineering, vascularization, immune incorporation, organ-on-chip technologies, spatial multi-omics, and computational biology, among other techniques, will significantly improve the physiological relevance of next-generation brain organoids. These diverse approaches will lead to improved tissue viability, cellular diversity, and functional maturity while integrating increasingly sophisticated molecular analyses and quantitative assessment of organoid fidelity. In brief, improved organoid systems are expected to narrow the gap between simplified in vitro models and the complex processes of the developing human brain, thereby expanding opportunities for neurodevelopmental research, disease modeling, and drug development.

## Brain Assembloids: Modelling Inter-regional Communication and Neural Circuit Assembly

### Concept and Historical Development

While traditional brain organoids have substantially advanced our knowledge of human neurodevelopment, region-specific organoids model individual brain regions in isolation, meaning these models do not recapitulate the complex interactions among anatomically and functionally distinct brain regions that occur during brain development. To address the limitations of conventional organoids, Birey et al. 2017 introduced the concept of brain assembloids, which are generated by fusing independently patterned, region-specific organoids to recapitulate the main processes of brain development. Subsequent advances enabled the fusion of organoids from different brain regions, allowing researchers to examine phenomena such as inter-regional communication, neuronal migration, and axon projection (Miura et al. 2022; Reumann et al. 2023).

As a major conceptual advance in human brain modeling, assembloids not only represent more sophisticated experimental models but also enable the integration of distinct neural tissues, facilitating the investigation of developmental processes in the brain associated with inter-regional communication. According to Miura et al. 2022; and Reumann et al. 2023; this technology shifted the focus of brain organoid studies from brain regions to higher-order neural circuits and their communication.

### Understanding Human Neurodevelopment Through Assembloids

Brain assembloids provide unprecedented opportunities to study developmental processes that could not previously be readily investigated in the human brain. For example, Birey et al. 2017 combined dorsal and ventral organoids and demonstrated that ventrally derived GABAergic interneurons derived from ventral organoids can tangentially migrate through the cortex, form synapses, and contribute to cortical circuit assembly.

More recent studies have used assembloids to investigate complex regional interactions, including corticothalamic interactions, corticostriatal connectivity, corticohippocampal interactions, axon guidance mechanisms, and neural circuit assembly (Reumann et al. 2023). Recently, researchers used assembloids incorporating multiple brain regions to investigate long-range neuronal connectivity and activity-dependent synapse formation (Kim et al. 2025).

### Disease Modeling and Translational Applications

The capacity to model functional communication between distinct brain regions has substantially expanded the applications of assembloids to various neurological and neuropsychiatric conditions. There are several disorders characterized not only by pathological features in specific neuronal populations but also by disrupted inter-regional connectivity within the brain. This suggests that assembloids are much better suited to studying medical conditions in which circuit dysfunction is a key pathogenic mechanism.

The study of patient-derived assembloids has provided important insights into abnormal interneuron migration and cortical circuitry in autism spectrum disorder and schizophrenia. Building on the pioneering work of Birey et al. 2017; and Reumann et al. 2023 further developed it. In the same manner, cortico-striatal and midbrain assembloid models have facilitated investigation of basal ganglia circuitry in Parkinson’s disease (PD), following the work of ventral midbrain organoids by (Luo et al. 2016) and the advancements in differentiation protocols established by (Fiorenzano et al. 2021). More recently, assembloid systems have also enabled investigation of aberrant neuronal synchrony in epilepsy and long-range network degeneration in Alzheimer’s disease (AD). Collectively, these studies have shown that transplantation into the host brain promotes neuronal maturation and functional integration (Miura et al. 2022). Numerous studies using patient-derived genetic backgrounds have provided important insights into disease mechanisms and therapeutic strategies that are difficult to obtain from studies with isolated organoids.

### Current Limitations

While brain assembloids offer substantial advantages, they remain subject to many of the limitations associated with conventional organoid systems. According to (Miura et al. 2022; Reumann et al. 2023), fusion efficiency varies across studies, leading to increased inter-batch variability in organoids. This is compounded by incomplete neuronal maturation, limited vascularization and metabolic support, and the absence of systemic physiological inputs, which continue to influence neuronal development. In experimental systems, brain assembloids recapitulate some mechanisms of inter-regional communication but do not fully reproduce those found in the human brain. This indicates that results produced using assembloid systems should be interpreted with caution regarding developmental and disease-related processes, rather than treated as faithful representations of human brain circuits.

### Future Perspectives

Brain assembloids represent one of the fastest-growing areas of organoid research and are expected to continue evolving alongside new biomedical technologies. Incorporating vascular networks, endothelial cells, microglia, and other non-neuronal cell types is anticipated to yield increasingly physiologically relevant multicellular models compared with existing organoid models, more faithfully recapitulating neurovascular and neuroimmune interactions. Integration with organoid-on-chip technologies and microfluidic perfusion systems will also likely enhance viability, maturation, and reproducibility of the tissues used, thus further building upon advances achieved with vascularized organoid systems (Miura et al. 2022).

The integration of assembloid technology and emerging technologies, such as spatial transcriptomics, single-cell multi-omics, electrophysiology, or high-resolution live imaging, will provide new insights into human neurodevelopment from molecular, cellular, and functional perspectives (Reumann et al. 2023). More importantly, the application of artificial intelligence and machine learning methods will enable quantitative analysis of neuronal connectivity, developmental trajectories, and multimodal data, accelerating biological discovery in basic biology and therapeutic development. Finally, transplantation of patient-derived assembloids into laboratory animals may promote maturation and functional integration, thereby providing a powerful platform for investigating regenerative and personalized medicine.

## Organat the Interface of In Vitro and In Vivooids 

### Transplantation as a Bridge Between In Vitro and In Vivo Biology

The transplantation of human brain organoids into animal models represents one of the most important recent advances, as it has combined the experimental control of organoid systems with the physiological complexity of the living brain. The integration of controlled in vitro models with the complexity of the in vivo brain has established a hybrid experimental platform bridging in vitro experimentation and in vivo neurobiology (Mansour et al. 2018; Miura et al. 2022).

**Fig. 5:**
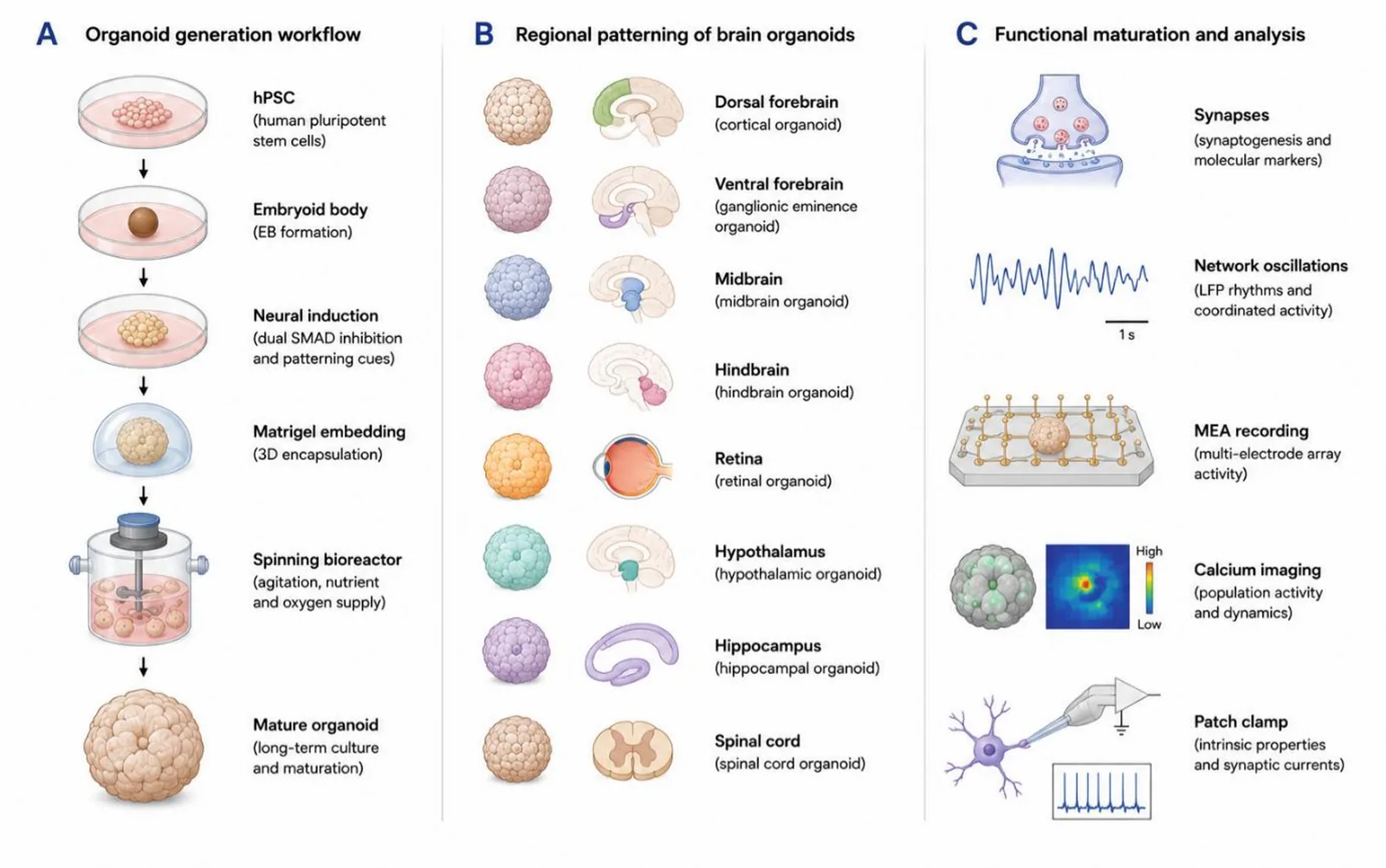
Generation and Functional Maturation of Brain Organoids. The chart above explains the technological method of obtaining of the human cerebral organoids from hPSCs and obtaining of some regions of the brain due to the directed differentiation of hPSCs. It also implies the systems used for the study of the process of organoids’ maturation. The following shows how brain organoids are created. First, pluripotent cells are differentiated into embryoid bodies (EBs), precursor neural structures and organoids by means of neural induction, embedding of ECM and culturing in the biotechnological systems. Secondly, by controlling signaling pathways organoids from the different brain regions and types of nervous tissue can be obtained. Lastly, different methods of maturation measurement were discussed (synapses construction, neuronal networks activity, multi-electrode measurements, calcium imaging, patch-clamp methodology) to assess the maturity of the organoids. Now organoids can be useful to study human dev. and modeling of neurodegenerative disorders. However, there is need in improvement of these technologies. The principle of this figure may combine the present successes in stem cell biology, developmental neurobiology, brain organoids construction, neurophysiology, bioengineering, and other new technologies, and methods related to the study of organoids or even artificial intelligence (Lancaster et al. 2013; Lancaster and Knoblich 2014; Qian et al. 2016; Pasca 2015; Kim et al. 2019; Mansour et al. 2018; Velasco et al. 2019; Chiaradia and Lancaster 2020; Lindenhofer et al. 2024). Abbreviations: EB, embryoid body; hPSC, human pluripotent stem cell; MEA, multi-electrode array; SHH, Sonic Hedgehog; WNT, Wingless/Integrated signaling pathway; BMP, bone morphogenetic protein; FGF, fibroblast growth factor; TGF-β, transforming growth factor-β

The earliest studies on transplantation indicate that cerebral organoids placed within the host brain survive for extended periods and undergo progressive host-derived vascularization via infiltration of host blood vessels. The process of vascularization significantly improves oxygen and nutrient delivery to cells, reduces necrosis, and promotes long-term neuronal survival compared with traditional cell culture methods (Mansour et al. 2018). More recent studies have demonstrated that vascularization promotes improved neuronal maturation during neuronal development and enhances the physiological relevance of transplanted organoids (Miura et al. 2022) (Fig. 5).

### Host Integration and Functional Maturation

Transplantation not only enhances physiological maturation but also provides evidence that human organoids can integrate into host neural circuits, both structurally and functionally. Once the organoids are implanted, they extend long-range axonal projections throughout the host brain but receive synaptic input from host neurons. Evidence from electrophysiological measurements indicates that the transplanted neurons can establish functional excitatory synapses, respond to various stimuli, and participate in local neural circuits (Mansour et al. 2018; Miura et al. 2022; Shen and Kokaia 2025).

Importantly, transplantation accelerates neuronal and glial maturation that would otherwise not be achieved in an in vitro system. Transplanted organoids show increased dendritic complexity, increased synaptogenesis, better electrical properties, enhanced astrocyte maturation and abundance, as well as improved oligodendrocyte maturation as compared to organoids of the same age grown entirely in vitro (Gordon et al. 2021; Miura et al. 2022; Lindenhofer et al. 2024). Additionally, infiltration by host-derived vasculature and glial cells provides organoids with new physiological cues absent in conventional in vitro organoid cultures.

All of these advances have substantially increased the translational relevance of brain organoids, enabling the study of neuronal survival, axon elongation, neural circuit formation, and function within living neural tissue. As a result, the significance of transplantation studies has dramatically increased because they contribute to the transition from disease modeling toward regenerative neuroscience in medicine.

### Applications for Disease Modeling and Regenerative Neuroscience

The combination of patient-derived organoids and in vivo transplantation provides powerful experimental systems for investigating neurological disorders under biologically relevant conditions. Organoids allow scientists to study human neurons over extended periods and investigate neuronal survival and maturation, as well as network development, while preserving patient-specific genetic backgrounds of nerve cells. These systems are particularly valuable for modeling neurodevelopmental and neurodegenerative disorders, brain injuries, and neural cell regeneration.

In addition to advances in disease modeling, transplantation serves as an important experimental platform for assessing regenerative approaches using human stem cell-derived neural tissues. While the clinical application of transplanted organoids remains a long-term objective, long-term survival, host-derived vascularization, and successful integration into host neural tissue have raised hopes for future cell replacement therapies and personalized regenerative medicine (Miura et al. 2022).

### Remaining Biological and Translational Limitations

Despite these promising developments, several important biological and technical limitations remain in current brain organoid technologies. Functional integration varies widely across experiments, while neuronal maturation remains incomplete in humans. In addition, organoids placed into the brain still exhibit incomplete cytoarchitectural organization and insufficient long-distance connections, which are necessary to establish the native physiological environment for the organoids (Miura et al. 2022; Lindenhofer et al. 2024). Furthermore, the intrinsic differences between human and rodent host environments can make the interpretation of the results from transplantation experiments more difficult and can influence processes and outcomes of such experiments, including neuronal maturation, neural circuit formation, as well as behavioral integration (Miura et al. 2022; Lindenhofer et al. 2024).

The increasing functional integration of transplanted human neural tissues in experimental animals raises important ethical considerations. The need to assess experimental design, animal welfare, and ethical review becomes increasingly important as organoid complexity and biological complexity increase.

### Future Perspectives

Future developments in transplantation research are expected to integrate advances in bioengineering, tissue engineering, organoid-on-chip technologies, and AI-assisted computational approaches. These approaches are expected to enable the integration of transplantation with multimodal molecular profiling, creating new possibilities for studying human neurons within physiologically relevant in vivo environments. Thus, the future of transplantation studies will involve integrating transplantation with next-generation organoid technologies (Mansour et al. 2018; Miura et al. 2022; Shen and Kokaia 2025).

## Signaling Factors that Modify Brain Development and Their Use in Organoids

**Fig. 6:**
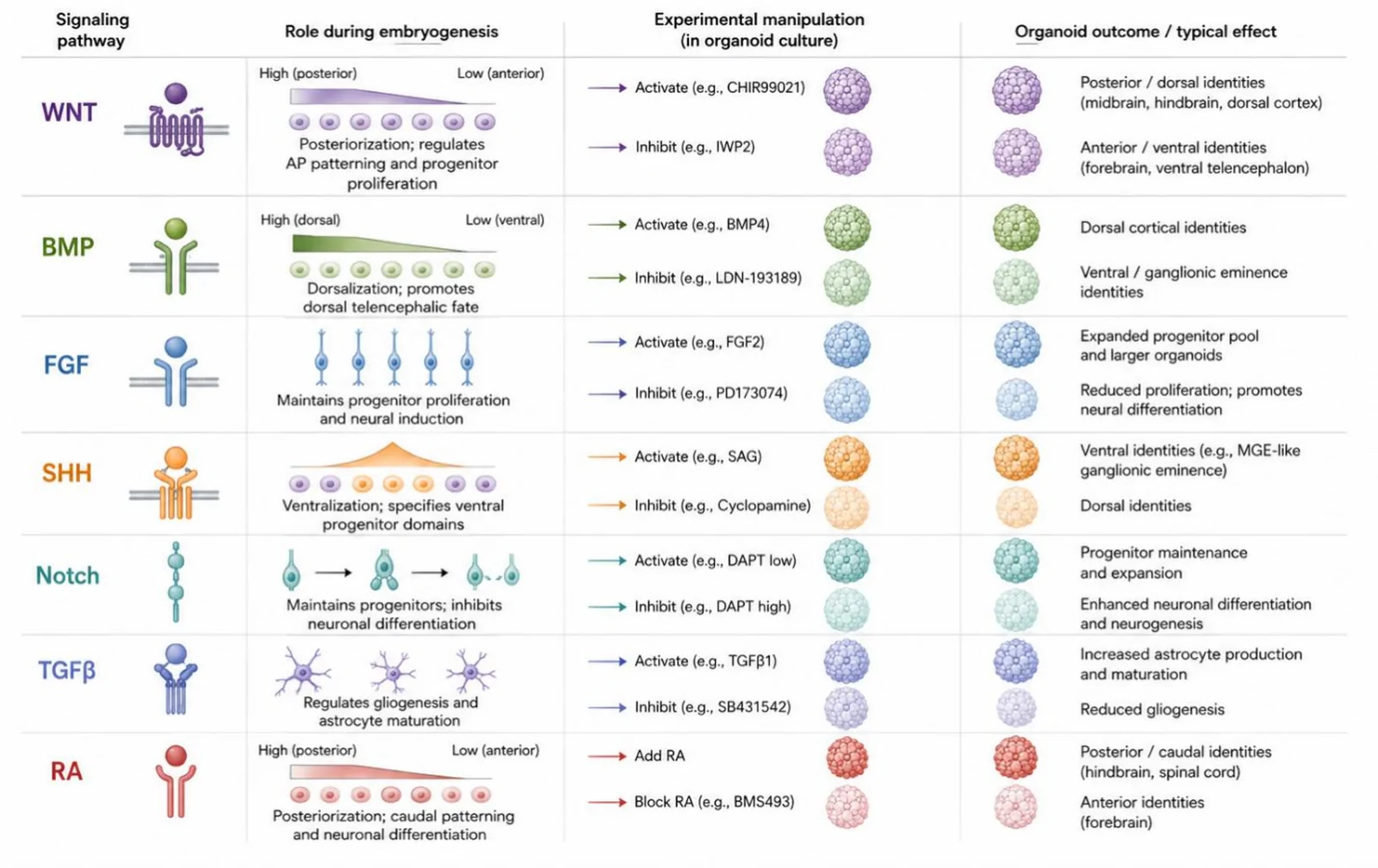
Major Developmental Signaling Pathways Used for Brain Organoid Patterning. The present figure summarizes the main development signaling pathways which control neural induction, regional patterning, progenitor maintenance, and cell lineage specification during human cognitive development. Moreover, it shows clearly how such pathways can be used to obtain region-specific human brain organoids.As for the pathways, they include the following ones: WNT, BMP, FGF, SHH, Notch, TGF-β, and RA signaling pathways. Each of them controls the development in the anterior/posterior, dorsal/ventral directions, expands the population of neural progenitor cells, and induces both neuronal and glial differentiation as well as regional specification.However, despite relative success in using the mentioned pathways for different brain organoid generation, it is still necessary to improve the quality of the brain organoids as such, as so far in most cases the organoids produced could not provide sufficient maturation time. The theoretical basis of the present figure is based on the results of many fundamental studies of developmental neurobiology applied to the processes of stem cells biology, directed differentiation, and brain organoid engineering (Eiraku et al. 2011; Lancaster and Knoblich 2014; Pașca et al. 2015; Qian et al. 2016; Xiang et al. 2017; Pașca 2015; Chiaradia and Lancaster 2020; Kim et al. 2019; Noack et al. 2023; Zenk et al. 2024). Abbreviations: BMP, bone morphogenetic protein; CHIR99021, glycogen synthase kinase-3 inhibitor; FGF, fibroblast growth factor; RA, retinoic acid; SAG, Smoothened agonist; SHH, Sonic Hedgehog; SMAD, Sma and Mad-related proteins; TGF-β, transforming growth factor-β; WNT, Wingless/Integrated signaling pathway

A major advantage of brain organoid technology is that it exploits many of the same developmental signaling pathways that regulate human embryonic brain development: it recapitulates many of the conserved developmental signaling pathways that govern human embryonic brain development. Therefore, precise temporal and spatial modulation of these conserved signaling pathways will allow researchers to achieve reproducible regional specification, tissue patterning, progenitor proliferation, and neuronal maturation in vitro. While these signaling pathways are well characterized, improvements in protocols for organoid differentiation primarily focus on optimizing the timing, duration, and dosage of signal combinations to enhance developmental robustness, reproducibility, and region-specific patterning (Chiaradia and Lancaster 2020; Qu et al. 2024, p. 1433111) (Fig. 6).

### TGF β (Transforming Growth Factor β)

During embryonic development, TGF-β signaling (including Nodal signaling) is essential for mesendoderm specification (Zhou et al. 1993; Schier 2003). Nevertheless, most organoid differentiation protocols inhibit TGF-β signaling, for example, through pharmacological inhibition using SB431542 in SFEBq aggregates (Kadoshima et al. 2013), human cortical spheroids (Paşca et al. 2015), and forebrain organoids (Qian et al. 2016; Xiang et al. 2017).

### BMP (Bone Morphogenetic Proteins)

In vivo, BMP signaling promotes non-neural ectoderm specification (Wilson and Hemmati-Brivanlou 1995) and contributes to specification of the dorsal neural tube and medial pallium (Liem et al. 1995; Subramanian et al. 2009) As in the case of TGFβ, BMP signaling is typically inhibited in the early stages of organoid development by the use of dorsomorphin or LDN 193,189 in hCS (Paşca et al. 2015) or cortical organoids (Xiang et al. 2017) to promote neuroectodermal differentiation while suppressing non-neural cell fates. Subsequent activation of BMP signaling at the developmental stage drives medial pallium (hippocampal) identities in organoid cultures (Watanabe et al. 2005; Eiraku et al. 2008).

### WNT Signaling

WNT signaling regulates multiple aspects of embryonic development. WNTs are involved in non-neural ectoderm induction (Mukhopadhyay et al. 2001), mesoderm induction (Rubenstein and Rakic 2013), midbrain–hindbrain specification (McMahon and Bradley 1990), caudal neural tube patterning (Rubenstein and Rakic 2013), cortical hem development (Subramanian et al. 2009), expansion of neuroepithelial progenitors (Chenn and Walsh 2002), and specification of deep-layer cortical neurons (Abu-Khalil et al. 2004). In vitro, WNT signaling is tightly regulated: early inhibition of WNT signaling (e.g., IWR 1endo in SFEBq or XAV 939 in cortical organoids) promotes anterior forebrain specification (Kadoshima et al. 2013; Xiang et al. 2017).

At later stages of differentiation, WNT is activated (e.g., with CHIR99021 and WNT3a) to promote neuroepithelial expansion, expansion of neural progenitor populations, and formation of the cortical hem or hippocampal primordium (Watanabe et al. 2005; Eiraku et al. 2008; Kadoshima et al. 2013; Lancaster and Knoblich 2014; Qian et al. 2016) In neocortical organoid slice cultures in neocortical organoids, transient WNT activation increases the formation of deep-layer neurons, whereas WNT inhibition increases the generation of upper-layer neurons (Qian et al. 2016).

### FGF (Fibroblast Growth Factor)

FGF signaling regulates multiple developmental processes in vivo, including mesoderm induction, telencephalic patterning, and establishment of the midbrain–hindbrain boundary. It induces mesoderm (Amaya et al. 1991), patterns telencephalon (Fukuchi-Shimogori and Grove 2001), and is involved in the midbrain-hindbrain boundaries (Crossley et al. 1996). The use of FGF2 has been extensive in organoids, promoting stem cell pluripotency and driving the continuous proliferation of neural progenitors (Lancaster et al. 2013; Paşca et al. 2015). Protocols for anterior tissues (telencephalic) have utilized FGF8 to guide differentiation toward forebrain types (Kadoshima et al. 2013).

### SHH (Sonic Hedgehog)

The role of Sonic Hedgehog signaling in embryonic development, particularly in neural tube specification, is considered important (Chiang et al. 1996). SHH agonists are widely used to induce ventral forebrain identities, including SFEBq, cortical organoids, and spheroid systems (Kadoshima et al. 2013; Lancaster and Knoblich 2014; Birey et al. 2017; Xiang et al. 2017). The importance of SHH signaling inhibition, for example, cyclopamine, which induces the production of dorsal features in cerebral organoid models, has also been proven (Lancaster et al. 2017).

### RA (Retinoic Acid)

Retinoic acid (RA) signaling activates the caudal part of the neural tube and promotes the development of progenitor and neural cells (Blumberg et al. 1997). When RA is used in organoids, it promotes neuronal differentiation and the maturation of intermediate progenitor cells (Lancaster et al. 2013; Xiang et al. 2017).

### ECM (Extracellular Matrix)

The factors of the extracellular matrix (ECM) affect the emergence of progenitor growth, shape and movement of cells, as well as their differentiation in vivo (Long and Huttner 2019). In the case of cerebral organoids, the introduction of aggregates that are derived from embryonic stem cells into the ECM (e.g. Matrigel) has become significant due to its role in stimulating NE formation as well as contributing to the process of cortical plate formation (Kadoshima et al. 2013; Lancaster and Knoblich 2014; Lancaster et al. 2017). The molecular underpinnings of differentiation protocols used for brain organoids involve the aforementioned signaling pathways. Recent advances focus on improving the timing and localization of signaling pathways to produce organoids that recapitulate specific brain regions. By introducing improved patterning methods in conjunction with bioengineering technology, maturation protocols and single-cell molecular standards, the accuracy of brain organoid development has significantly improved. Future development of protocols will likely combine regulated signaling during development with computational and quantitative approaches, based on human reference atlases, to improve reproducibility and clinical relevance.

## Applications in Disease Modeling and Therapeutics

**Fig. 7:**
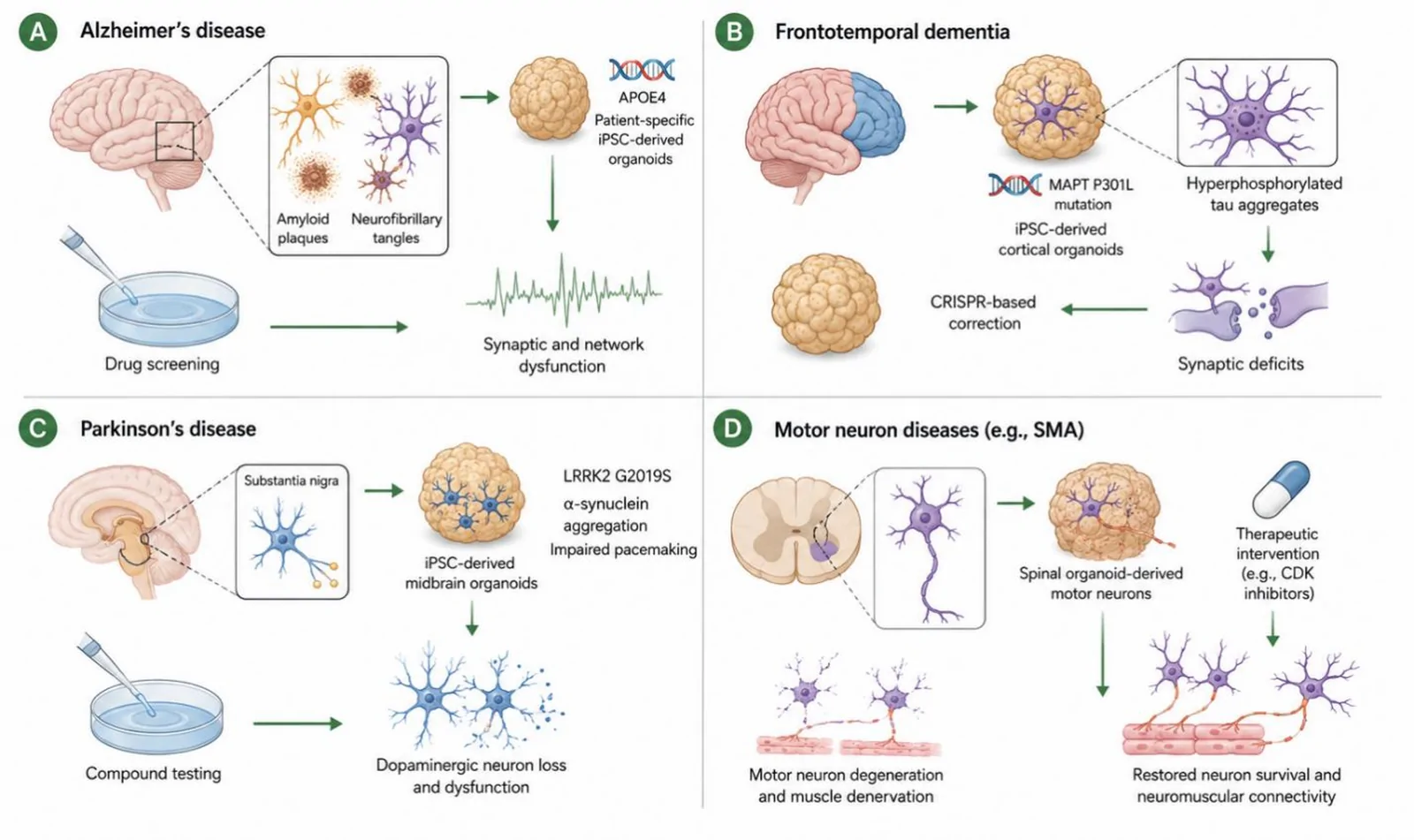
Brain Organoids for Disease Modelling and Therapeutic Discovery. This illustration depicts the various neurological disorders modeled using brain organoids obtained from patients. The image illustrates how brain organoids are applied in modeling the main neurological disease known to man including AD, PD, frontotemporal dementia, motor neuron disease, spinal muscular atrophy, and schizophrenia. Brain organoids mimic the important cellular, molecular, and functional aspects of the selected disorders. Even though brain organoids are expected to act as human-centered systems for the analysis of the mechanism of diseases as well as for the determination of potential therapeutic targets and estimation of possible treatments, the image demonstrates how organoids are developing as systems for modeling complex neurological disorders and the experimental treatment analysis. Both the image and the recent discovery in the fields of human genetics and genetic engineering, stem cells, organoid science, microphysiological cultures, genome editing, single-cell and spatial omics, and systems biology are discussed in this schematic (Lancaster and Knoblich 2014; Jo et al. 2016; Birey et al. 2017; Jourdon et al. 2023)

Brain organoids are being recognized as effective human platforms for investigating neurological and neuropsychiatric disorders that cannot be faithfully recapitulated in traditional animal models or 2D cultures (Fig. 7). Since organoids are capable of retaining patient-specific genetic backgrounds and recapitulating key features of human neurodevelopment, they have become among the most informative experimental platforms for investigating disease mechanisms, as well as genotype-phenotype correlations and therapeutic responses or therapeutic development. Recent technological advances such as region-specific organoids, vascularized organoid models, assembloids, advanced maturation strategies, and multicellular models have substantially expanded the capabilities of brain organoids while also highlighting the biological limitations that currently constrain their translational applicability in a clinical context (Li et al. 2025; Liu et al. 2025).

Although disease-specific organoids have provided important mechanistic insights into neurological disorders, they should not be regarded as complete representations of disease pathology but rather as powerful platforms for mechanistic investigation. Aging, systemic immune responses, vascular dysfunction, hormonal effects, and long-term environmental influences contribute substantially to the pathogenesis of chronic neurological disorders. As a result, organoids should be integrated with evidence from animal models and clinical studies to provide a comprehensive understanding of diseases (Velasco et al. 2019; Noack et al. 2023; Zenk et al. 2024).

Notably, successful recapitulation of disease-associated cellular phenotypes does not mean that these systems fully recapitulate disease progression as it progresses. Various neurodegenerative diseases include complications such as aging, systemic inflammation, vascular dysfunction, and complex systemic physiological interactions, all of which have not yet been faithfully recapitulated in current-generation organoid models.

### Alzheimer’s Disease

Although modeling age-associated neurodegenerative diseases using embryonically derived cells presents inherent limitations, previous studies have demonstrated that cerebral organoids can nonetheless recapitulate significant aspects of AD. An early landmark study by Choi et al. generated human neural stem cells or developed a three-dimensional culture system using human neural stem cells expressing lentiviral constructs with either β-amyloid precursor protein (APP) or presenilin-1 (PSEN1) familial AD-associated variants (FAD) in Matrigel to create a 3D model of AD pathology (Choi et al. 2014).

Although this model did not employ conventional organoid protocols, these three-dimensional cultures developed extracellular β-amyloid plaques in addition to intracellular aggregates of phosphorylated tau pathological hallmarks that are rarely reproduced simultaneously in traditional 2D neuronal cultures and, more importantly, not in most animal models of AD. Their results support the idea that rodent models incompletely recapitulate human neurodegeneration and that 2D cultures, in general, lack the tissue architecture and cellular complexity needed to support the aggregation of pathogenic proteins (Kovacs et al. 2013, 2017; Xekardaki et al. 2014).

Additionally, this model provided a valuable platform for therapeutic screening: inhibition of glycogen synthase kinase 3 (GSK3) led to significant reductions in both plaque burden and tau pathology, supporting hypotheses that had previously been tested only in animals (Phiel et al. 2003; Jaworski et al. 2011). At a time when so many compounds that were successful in initial animal testing failed in later trials, these results highlight the translational potential of organoid-based drug screening (Doody et al. 2013, 2014; Salloway et al. 2014).

Following this work, Lee et al. established cortical organoids from sporadic AD patient-derived iPSCs to mitigate potential issues associated with transgene overexpression (Lee et al. 2016). Cortical organoids generated from these more physiologically relevant conditions developed robust β-amyloid plaques.

Most importantly, their disease-associated phenotypes were ameliorated by inhibition of β-secretase (BACE1) or γ-secretase, supporting their use as therapeutic screening platforms; however, the protease inhibitors were less effective in 3D organoid cultures than in the 2D cultures they ran in parallel. The reduced therapeutic efficacy with the protease inhibitors reinforced the idea that diffusion constraints or tissue architecture in organoids can more closely recapitulate the native tissue microenvironment for drug testing. They also comment on patient-to-patient variability: five AD organoid lines derived from patients with similar plaque loads showed different outcomes in secretase inhibition testing. Proteomic analyses revealed that variation in the abundance of specific proteins, such as clathrin, correlated with functional outcomes, suggesting that organoids may be valuable platforms for personalized therapeutic screening to evaluate how patient-specific molecular backgrounds influence their pharmacological response (Lee et al. 2016; Raja et al. 2016).

Additionally, the same inhibitors used in the original 3D model by Choi et al. reduced neurofibrillary tangles, supporting the utility of distinct organoid models in drug discovery. A third group confirmed these observations, demonstrating that organoids derived from sporadic AD patients consistently recapitulate β-amyloid plaque formation, neurofibrillary tangles, and endosomal abnormalities observed previously in both mouse models and human patients (Cataldo et al. 2000, 2001; Raja et al. 2016).

Even organoids derived from healthy individuals showed low basal levels of amyloid and tau, similar to the subclinical deposits often found in neurologically normal humans (Bennett et al. 2006; Kovacs et al. 2013, 2017; Xekardaki et al. 2014). This ability to generate not only pathology, but also physiological states suggests a promising strategy for studying early disease initiation.

Building on this work, the same group used CRISPR-engineered APOE2/3/4 isogenic lines to investigate how the APOE genotype—the most robust genetic risk factor for AD—modulates cellular vulnerability (Lin et al. 2018). Using neuron populations derived from APOE4 individuals, they demonstrated that alterations in lipid metabolism and immune signaling pathways were mediated in part by NF-1, AP-1, and NF-κB signaling, and that these changes were accompanied by increased gene expression associated with neuronal synapse biology and elevated γ-secretase-mediated amyloid-β42 secretion.

By introducing APOE4 microglia into organoids derived from APP-duplicated iPSCs, the researchers observed phagocytic dysfunction and morphological changes, along with elevated levels of soluble TREM2. This implies that APOE4 contributes to impaired microglial function, which is involved in the development of amyloid deposits. Multicellular co-culture systems illustrate the capacity of organoids to combine genetically similar cell types to investigate the roles each cell type plays in disease.

Using the idea of multicellular complexity as a foundation, Park et alia developed an innovative microfluidic platform to introduce large quantities of microglia into mixed cultures of astrocytes and neurons in three-dimensional environments while recapitulating key features of AD pathology such as accumulation of beta-amyloid deposits, phosphorylation of the tau protein, and neuroinflammatory processes observed in the human brain (Park et al. 2018). Their platform enabled investigation of the chemokine-mediated recruitment of microglia by CCL2, CCL5, and CX3CL1, as well as the cytokines released by microglia, IL-6, IL-8, and TNF-α, within a physiologically relevant three-dimensional microenvironment.

These studies demonstrated that microglial migration and ensuing neuronal loss depend on the TLR-4 and IFN-γ systems, demonstrating the ability of organoids to provide an experimentally tractable yet physiologically relevant platform for studying neuron-immune communication and testing the effects of immunomodulatory treatments.

Recent advances have substantially improved the physiological relevance of organoid models for AD by integrating multiple brain-resident cell types and establishing complex microenvironments. The implementation of vascularized organoids, co-cultures with microglia, and multicellular platforms has enabled investigation into neuropathological mechanisms of neurovascular function, blood-brain barrier integrity, and neuroimmune communication in the presence of traditional amyloid- and tau-based pathology. Patient-specific iPSC-derived organoids derived from iPSCs enable investigation of inter-individual variability in disease mechanisms and therapeutic approaches. Although these advancements allow brain organoids to serve as complementary translation platforms in drug discovery, faithfully modeling aging and late-stage neurodegeneration remains a major challenge.

### Frontotemporal Dementia

Beyond AD, brain organoids are increasingly providing insight into pathogenic mechanisms in other neurodegenerative diseases. In frontotemporal dementia, iPSC-derived organoids exhibited significantly increased tau phosphorylation in those with a Tau P301L mutation, while CRISPR-mediated insertion of a non-cleavable p35 variant partially rescued synaptic deficits (Seo et al. 2017).

Brain organoids have recently gained prominence as useful tools for research into neurological and psychiatric diseases linked to cortical development and neural circuit formation (Birey et al. 2017; Chiaradia and Lancaster 2020). Besides the case of schizophrenia, patient-derived organoids have been employed to study autism spectrum disorder, bipolar disorder, Rett syndrome, and other related neuropsychiatric illnesses (Mariani et al. 2015; Birey et al. 2017; Miura et al. 2022). Additionally, new methods that combine region-specific organoids into assembloids enable the study of processes such as interneuron migration, cortical connectivity, and long-distance circuit formation (Birey et al. 2017; Miura et al. 2022). The advantage here is that such approaches are well-suited to the assessment of patient-specific treatments, since the only problem is the incomplete development of organoids (Chiaradia and Lancaster 2020; Miura et al. 2022).

### Parkinson’s Disease

For the study of PD, region-specific organoid differentiation strategies are being applied. In one study by Kim et al. (2019), LRRK2 mutations were introduced into midbrain organoids, resulting in reduced expression of dopaminergic neuronal markers, consistent with dopaminergic neurodegeneration and co-localization of the phosphorylated S129 form of alpha-synuclein with endosomes (Kim et al. 2019).

Comparative transcriptomic analyses of sporadic brain PD tissues revealed strong molecular similarity relative to two-dimensional cultures, while gene silencing of TXNIP, a key regulator in the LRRK2-mediated transcriptome, significantly reduced synuclein aggregation, underscoring the potential of these systems to identify druggable targets.

Similarly, researchers Smits et al. (2019) generated iPSCs from patients with the G2019S mutation to create midbrain organoids that exhibit robust dopaminergic neuronal differentiation and physiologically relevant pacemaker activity (Smits et al. 2019). Interestingly, the authors reported that in both patient cases, including the one without the mutation, the number of dopaminergic neurons produced was lower than in their healthy controls.

Ventral midbrain organoids have moved from proof-of-concept models to more mature, standardized systems for investigating PD pathogenesis. The differentiation methods have improved the consistency of generating dopaminergic neurons and have enabled investigation of neuronal development, including α-synuclein aggregation, mitochondrial impairment, lysosomal dysfunction, and neuroinflammation. Jo et al.’s study (2016) demonstrated the generation of functional human midbrain organoids containing various dopamine-producing neurons (Jo et al. 2016). This technology was later improved, as reported by Fiorenzano et al. 2021; with enhanced efficiency and reproducibility. Recent technologies in the field have improved cellular structures, electrophysiological development, and the consistency of outcomes, facilitating mechanistic studies and therapeutic discovery for PD treatment (Kajtez et al. 2025).

Notably, these advanced midbrain organoids are frequently used alongside single-cell transcriptomics, spatial profiling, and transplantation techniques to assess developmental fidelity and functional maturation. However, current models still fall short of attaining physiological aging, vascularization, and neuro-immune interactions, indicating that they recapitulate key biological features of Parkinson’s disease but not the full spectrum of disease pathogenesis.

### Motor Neuron Diseases

Brain organoids have also been used to investigate motor neuron diseases. Kawada et al. (2017) developed a microfluidic platform to generate aligned axon bundles from neural spheres enriched with spinal motor neuron precursors (Kawada et al. 2017). A major advantage of this approach was its ability to produce directional axon bundles that fired synchronously, enabling investigation of axonal injury caused by hydrogen peroxide-induced oxidative stress.

By employing protocols incorporating graded caudalizing and ventralizing signals, spinal organoids resembling rosettes were created, supporting the proliferation and maturation of both neural stem cells and motor neurons, and forming functional neuromuscular junctions using mouse matrix. Subsequently, Grass et al. (2024) applied recently developed differentiation protocols to model spinal muscular atrophy (SMA) and demonstrate that, although ISL1-positive neurons are formed from patient-derived cells, they still undergo degeneration. Moreover, CDK inhibitors were shown to reverse SMN-deficient cell lines. Collectively, these findings demonstrate that organoids can be engineered to study region-specific disease vulnerability on the neural axis (Grass et al. 2024).

### Psychiatric Disorders

Recent studies have shown that organoids are valuable for elucidating neurological conditions across neurodevelopmental and neurodegenerative disorders. For instance, in the case of schizophrenia, certain cerebral organoids that were derived from patients showed delayed maturation of neurons and aberrant FGFR1 nuclear signaling during early stages of corticogenesis (Stachowiak et al. 2017).

In complementary studies of 16p13.11 microduplication, correlations were identified between reduced cortical thickness on MRI, smaller patient-derived organoids, and altered progenitor division modes—all of which were reversible when NF-κB signaling was pharmacologically activated (Johnstone et al. 2019).

Notwithstanding these advances, disease-specific brain organoids should be viewed primarily as platforms for mechanistic discovery rather than as comprehensive models of disease pathophysiology. The majority of neurological diseases are influenced by aging-related processes, systemic immune responses, vascular defects, endocrine influences, and long-term environmental interactions; these factors cannot all be recapitulated in existing organoid technologies. As a result, findings from organoid studies should be interpreted alongside additional evidence from animal studies, human genetics studies, clinical studies, and postmortem tissue analyses. Progress in vascularization, advanced maturation strategies, bioengineering, and computing technologies are likely to make organoid technologies more clinically translatable.

Using disease-specific brain organoids, researchers can investigate human neurological diseases in a unique way, addressing questions that cannot be readily investigated in patients or animals. As researchers continue to develop region-specific differentiation, multicellular co-culture systems, vascularization, assembloid technology, and single-cell multi-omics, the biological and physiological fidelity and translational utility of organoids will continue to improve. However, it should be recognized that currently developed organoid models may still be treated as adjunctive human-specific tools that reproduce some facets of disease pathophysiology but not the disease in its entirety. It is likely that integrating organoids with clinical genomics, computational modeling, and other experimental tools will yield their greatest translational impact.

## Emerging Technologies and Future Frontiers

**Fig. 8:**
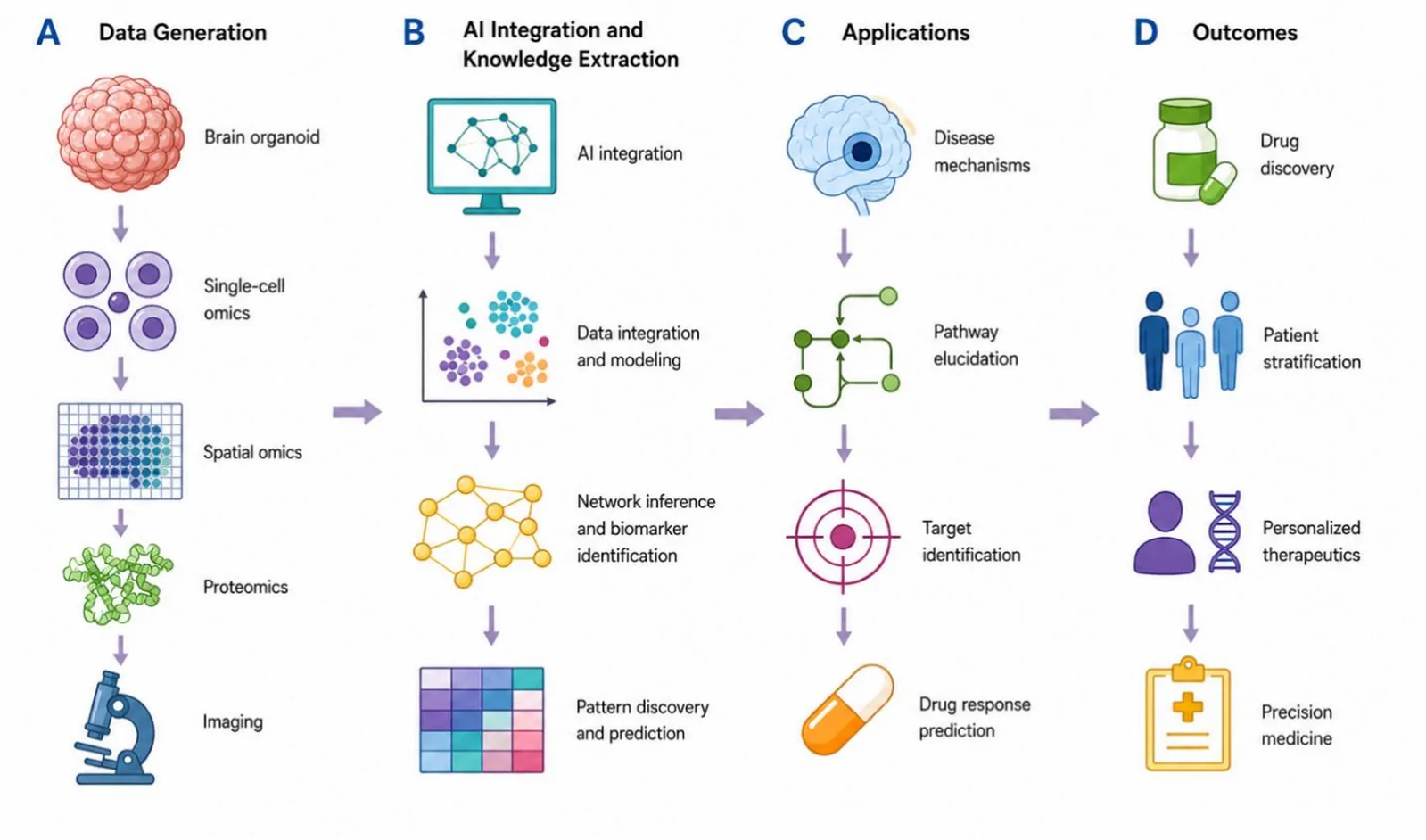
AI-Enabled Multi-Omics Integration for Brain Organoid Research. The graphic shows how transcriptomics, spatial omics, proteomics, metabolomics, imaging, and electrophysiologicaldata produce brain organoids through AI-ML. AI based computing methods aid in discovering developmental routes, disease pathways, molecular derivatives, and facilitating methods of dealing with patients under precision medicine. In general, the image emphasizes the importance of the application of AI in computational biology for transforming brain organoid research into a quantitative, data-driven discipline and guiding experimental validation of the research outcomes. The figure is developed by combining and utilizing current technological innovations in the fields of human genetics, functional genomics, stem cell biology, organoid and microphysiological systems, genome editing, single-cell and spatial omics, systems biology, as well as AI (Cuomo et al. 2020; He et al. 2023; Noack et al. 2023; Zenk et al. 2024; Camps et al. 2025)

Recent advances in multi-omics technologies, advanced imaging techniques, and computational biology have transformed the study of brain organoids. Brain organoids are no longer merely descriptive models of development; they enable quantitative comparisons with primary human tissues at the single-cell level and provide high-resolution analyses of developmental fidelity, cellular diversity, and molecular changes associated with diseases (Fig. 8). The use of data from the transcriptome, epigenome, proteome, metabolome, and spatially resolved omics approaches has transformed organoid science into a quantitative systems biology discipline and provides mechanistic insights into the molecular basis of human neurodevelopment and neurodegenerative conditions. The use of artificial intelligence and machine learning has further advanced the field by enabling analysis of multidimensional datasets in novel ways and the identification of complex biological patterns that would otherwise remain undetected.

### Deciphering Cellular Diversity and Developmental Trajectories

Recent breakthroughs in high-throughput multi-omics have transformed brain organoid research, providing unprecedented insights into cellular and molecular mechanisms during human neurodevelopment. While bulk RNA-seq has enabled global characterization of transcriptional changes during organoid differentiation, scRNA-seq has substantially advanced the field by resolving many differences at the cellular level (Cuomo et al. 2020; Lu et al. 2021).

Recently, advanced methodologies—including RNA sequencing (scRNA-seq), spatial transcriptomics, scATAC-seq, chromatin accessibility profiling, proteomics, and metabolomics—have been used to generate comprehensive molecular atlases of brain organoid development. The advent of these multimodal techniques has given scientists many opportunities to discover developmental trajectories, identify previously unrecognized neuronal populations, and investigate gene regulatory networks that govern corticogenesis in humans. The simultaneous use of several methods has substantially advanced understanding of cell development processes (Noack et al. 2023; Zenk et al. 2024, 2024).

Notably, single-cell transcriptomic analyses have enabled the evaluation of developmental fidelity by directly comparing organoids with primary human brain tissue. Studies comparing data from organoids have shown how the protocols now applied faithfully recapitulate multiple aspects of the fetal human brain’s development and neuronal differentiation, even as they also point out major limitations, such as incomplete neuronal maturation after differentiation as well as the limited generation of mature astrocytes and oligodendrocytes and the inability to establish functional neurovascular networks or to recapitulate higher-order tissue architecture. In contrast, it has enabled quantitative benchmarking of organoid fidelity (Noack et al. 2023; Zenk et al. 2024, 2024).

Transcriptomic profiling is now routinely integrated with proteomic and metabolomic profiling, providing complementary functional insights that cannot be obtained from gene signatures alone. In proteomics studies, multiple signaling pathways and protein interactions were found that are associated with disease phenotypes in patient-derived brain organoids. In turn, the metabolomic profiling revealed several metabolic alterations associated with neural differentiation, neuronal maturation, and stem-cell fate decisions. Consequently, the integration of transcriptomic, proteomic, and metabolomic information has provided an opportunity to obtain a comprehensive understanding of the molecular mechanisms governing normal human brain development and the development of neurological disorders (Bi et al. 2021; Simintiras et al. 2021; Wang et al. 2021; Noack et al. 2023).

Advances in imaging technologies have enabled increasingly detailed analyses of organoids’ structure and function. Advances such as tissue clearing, light-sheet microscopy, fluorescence microscopy, live-cell imaging, and genetically encoded fluorescent markers have enabled visualization of three-dimensional organoid development in real time with exceptional spatial and temporal resolution. Integrating multi-omics with advanced imaging enables multimodal characterization on a multimodal platform (Ueda et al. 2020; Giandomenico et al. 2021; Shah 2024).

The integration of single-cell multi-omics, imaging, and computational analysis approaches has transformed brain organoid research from descriptive biology into quantitative systems biology. These methods have substantially advanced our understanding of the molecular mechanisms of corticogenesis and enable us to assess organoid fidelity, reproducibility, and their translational potential (Noack et al. 2023; Zenk et al. 2024).

### Benchmarking Organoid Fidelity

One of the most significant recent advances in brain organoid research is the establishment of quantitative benchmarking strategies that enable objective assessment of brain organoid fidelity relative to the developing human brain. Now, using single-cell transcriptomics, spatial transcriptomics, epigenomic profiling, proteomics, and computational analysis, researchers can directly compare organoid cells with a reference atlas derived from human fetal brain tissues. The multidimensional nature of these approaches provides a robust framework for evaluating developmental stage, regional identity, and cellular composition, as well as cellular differentiation trajectories, while distinguishing true biological variation from technical variability (He et al. 2023; Noack et al. 2023; Zenk et al. 2024).

In addition to characterizing cellular composition, benchmarking has greatly improved our understanding of modern brain organoid systems. Comparison results show that current organoids faithfully recapitulate aspects of human brain development at the molecular and cellular levels (e.g., neuronal lineage specification and the identification of region-specific progenitor populations). Yet benchmarking also has limitations. For example, it cannot fully account for persistent immature neuronal populations, limited representation of mature astrocytes and oligodendrocytes, limited vascularization, incomplete neuroimmune interactions, or limited long-range neuronal connectivity. This is why benchmarking is a necessary tool for identifying biological limitations that must be solved by optimization of differentiation protocols, bioengineering, and maturation (Noack et al. 2023; Zenk et al. 2024).

It’s important to note that the advent of quantitative benchmarking has substantially improved quality control in brain organoid research by providing metrics for differentiation efficiency, reproducibility across laboratories, and protocol standardization. Beyond relying solely on morphological observations or a limited number of molecular markers of development and differentiation, the current benchmarking framework employs transcriptomic, epigenomic, proteomic, and spatial data to generate comprehensive molecular profiles of organoids. The multimodal nature of this approach to this technology enables comparison of results obtained in different laboratories, thereby facilitating protocol optimization and cross-study reproducibility (Noack et al. 2023; Camps et al. 2025; Schimelman et al. 2026).

Benchmarking has evolved beyond developmental biology applications in developmental biology and is an important component of translational research as well. Benchmarking provides an objective framework for assessing whether organoid models created for specific diseases faithfully recapitulate disease biology. Benchmarking aims to evaluate therapeutic responses and improve reproducibility in preclinical studies. Moreover, the unbiased assessment of organoid fidelity is now seen as a prerequisite for regenerative medicine and clinical translation, as reproducibility, reliability, and biological consistency are essential to these processes. As reference atlases and comparative datasets develop, the need to benchmark and optimize the standardization of innovative brain organoid production technologies will increase (Zenk et al. 2024; Camps et al. 2025).

So, quantitative benchmarking has helped advance brain organoid studies by transforming qualitative observations into quantitative assessments. This methodology employs multi-omics approaches and advanced computing technologies to provide robust quantitative metrics of maturity level and to identify the biological limitations that differentiate organoids from a developing human brain. However, to improve reproducibility, expand the use of organoids in neuroscientific studies, and establish them as reliable human model systems, it is important to continue improving benchmarking processes, standard reference atlases, and standardized computational analysis pipelines (Noack et al. 2023; Zenk et al. 2024; Camps et al. 2025).

### AI-Enabled Biological Discovery

Artificial intelligence (AI) has rapidly emerged as one of the most transformative computational technologies in brain organoid research, enabling the investigation of high-dimensional biological datasets that extend beyond conventional statistical approaches. Today’s machine-learning and deep-learning models enable researchers not only to automate data processing but also to extract biologically meaningful information from multidimensional datasets (He et al. 2023; Shah 2024).

The quantitative analysis of brain organoids has greatly benefited from AI-based image analysis, which enables large-scale assessments of brain organoid structures, tissue architecture and axonal organization, tissue morphogenesis, cellular organization, and electrophysiological behavior. AI techniques make it possible to reduce observer bias in the researcher’s observations, improve analytical reproducibility, and enable large-scale quantitative analyses. As a result, image processing platforms developed on the basis of AI technologies have established themselves as effective means for automated characterization of brain organoids (Shah 2024).

In addition to image analysis, AI is accelerating biological discovery through multimodal data integration. Machine learning algorithms can identify novel cell populations, reconstruct developmental trajectories, infer gene regulatory networks, predict lineage relationships, and integrate transcriptomic, epigenomic, proteomic, metabolomic, imaging, and electrophysiological data into a unified systems-level framework. Integrative approaches provide a more comprehensive understanding of the molecular mechanisms of human corticogenesis and, at the same time, accelerate hypothesis generation and facilitate interpretation of complex developmental processes (He et al. 2023).

Crucially, AI has made significant advances in quantitatively measuring organoid reproducibility and developmental fidelity. Incorporating organoid datasets with reference atlases of the developing human brain enables objective quantification of regional specification, cellular composition, and developmental maturity by distinguishing biological variation from protocol-related technical variability. These methods have been successfully applied to optimize differentiation protocols from organoid quality benchmarking and to improve reproducibility across labs (He et al. 2023; Noack et al. 2023; Zenk et al. 2024).

AI extends organoid research beyond descriptive analyses and is transforming translational neuroscience over time. The use of modern machine learning approaches enables the identification of molecular biomarkers associated with specific neurological disorders, the prediction of treatment response, the classification of organoid phenotypes in patients, and the identification of the most promising therapeutic targets. AI-driven computational frameworks hold considerable promise for precision medicine by integrating diverse data from organoid samples collected from individual patients and constructing predictive disease models of neurological diseases, thereby significantly accelerating drug discovery in preclinical research (Noack et al. 2023; Shah 2024).

AI, as a whole, is transforming brain organoid research from descriptive biology to predictive, data-driven systems biology. By integrating advanced imaging technologies, multi-omics-based methodologies, and computational modeling, AI enables exploration of human neurodevelopment, elucidates disease mechanisms, and enhances the reproducibility and translatability of brain organoids. Thus, further development of computational approaches will require rigorous experimental validation.

### Computational Integration and Remaining Challenges

Despite the significant advances enabled by multi-omics technologies and AI, several computational challenges continue to constrain the full potential of brain organoid research. Multi-omics datasets from various labs exhibit substantial variability, making direct cross-study comparisons challenging. Moreover, integrating transcriptomic, epigenomic, proteomic, metabolomic, imaging, and electrophysiological datasets into a single biological framework remains a major computational challenge. Thus, standardization of analytic pipelines, reference atlases, and robust quality-control frameworks is essential for achieving reproducibility in organoid research and for enabling valid comparisons between organoid systems (Noack et al. 2023; Camps et al. 2025).

Another major challenge in using AI-generated predictions is interpreting these predictions. Although machine learning methods can identify complex biological patterns, computational predictions do not establish causal biological relationships and therefore require experimental validation. Increased use of AI in organoid research also highlights the need to develop explainable artificial intelligence (XAI) to provide mechanism-based interpretations of predictions, rather than relying on incomprehensible “black-box” models, thereby improving confidence in AI-based findings and facilitating regulatory acceptance (Chen et al. 2024; Trapnell 2024).

Future developments are expected to center on brain organoids, as a continuous integration of computational biology, bioengineering, advanced imaging, and organoid technology will further establish them as robust, quantitatively validated models of human neurodevelopment and neurological diseases. The future of brain organoid technology relies not only on the development of new computational techniques but also on integrated multidisciplinary approaches that integrate multimodal biological datasets, computational pipelines, open-access reference resources, and experimental validation strategies.

With the continued increase in brain organoid complexity, both structural and functional, advances in computational methodologies will need to be closely followed by continued consideration of the ethical implications of their use. The increasingly complex organoid systems, especially those capable of more advanced neural functions, raise new concerns about the regulation and interpretation of functional activity data from organoid use. Addressing ethical questions alongside advances in technology is critical to the safe and responsible application of new brain organoid systems for clinical and research use.

**Fig. 9:**
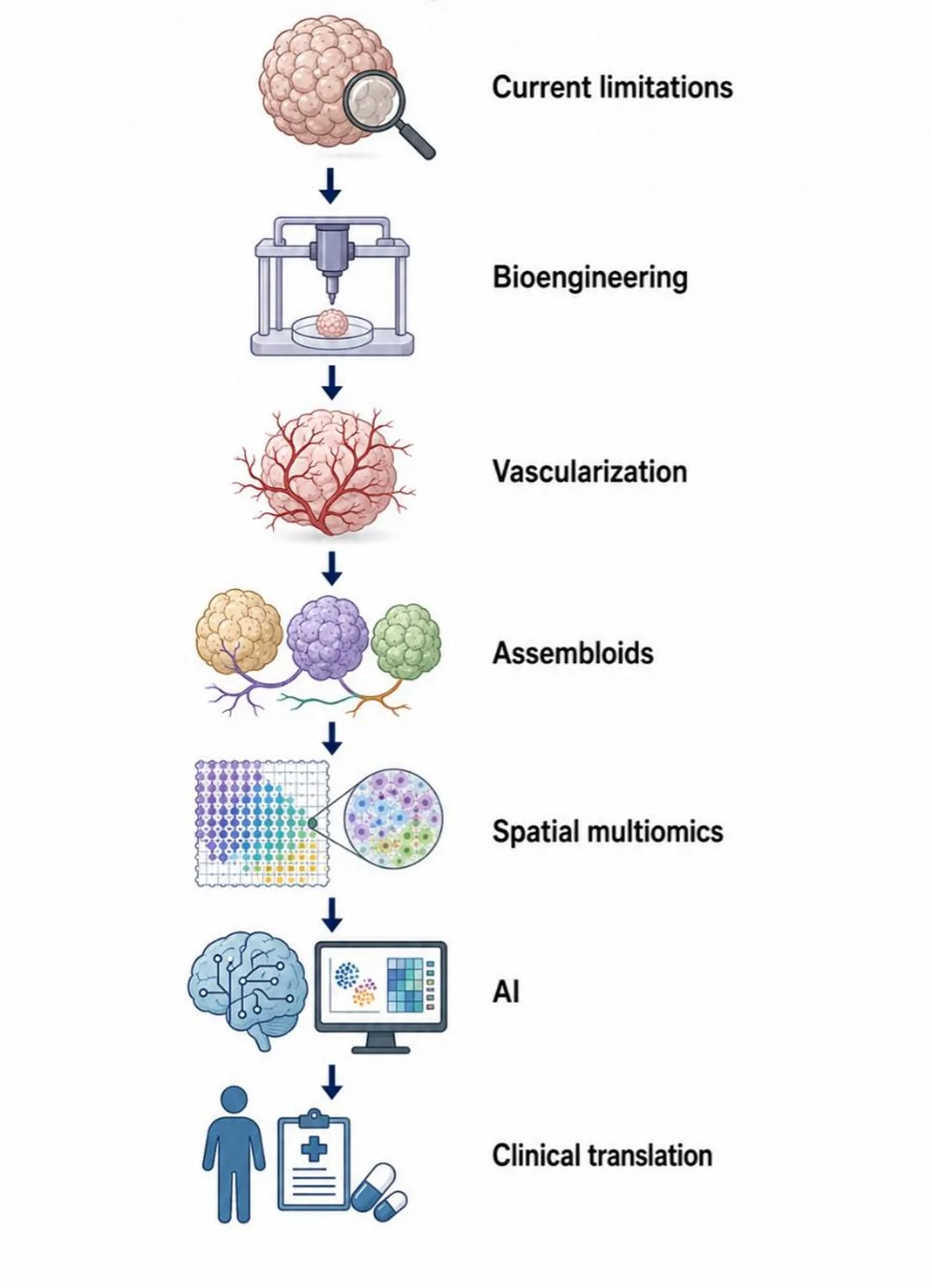
Future directions in brain organoid research. The figure depicts the new strategies that could enhance the biological fidelity and translational potential of brain organoid systems. Taken together, new achievements in bioengineering, vascularization, assembloid technology, spatial multi-omics analysis, AI-driven computational approaches, and clinical translation are expected to drive the next generation in the field of human brain organoids. The figure demonstrates the role of the technologies mentioned in developmental fidelity, functional maturation of organoids, disease modeling, drug discovery, and precision medicine. The figure is developed through the integration of the latest advances in genetic engineering, functional genomics, stem cell technology, organoid systems and microphysiology, genome editing, single-cell omics and spatial omics, systems biology, and AI (Miura et al. 2022; Noack et al. 2023; Reumann et al. 2023; Zenk et al. 2024; Camps et al. 2025)

Taken together, these technological and computational developments make brain organoids valuable not only as descriptive experimental models but also as human-specific, quantitatively evaluated systems, thus advancing the fields of developmental genetics, disease modeling, and personalized medicine. Progress will further rely on the integration of bioengineering practices, standardized atlases, multimodal validation strategies, and clarity (Fig. 9).

## Limitations and Ethical Considerations

Although neural organoids have been widely used in vitro neuroscience, they represent a balance between biological fidelity and experimental abstraction. The current state of the field points to both the substantial potential and the limitations of these models for understanding human brain biology. Organoids undoubtedly provide a new platform for interrogating issues related to human neurodevelopment, neuropsychiatric disorders, infectious pathology, and evolutionary biology in a 3-dimensional context. Nevertheless, it is worth remembering that organoids are biologically informed approximations of the biological systems they intend to model.

The limitations of brain organoids are crucial to biological interpretation, not just technical considerations. Variability in differentiation procedures, incomplete maturation, limited cell diversity, and absence of functional vascularization are among many factors that can influence cell composition, transcriptional stages, neuronal physiological parameters, and therapeutic responses. Therefore, the reproducibility of one organoid cannot be measured solely by shape similarity but also by quantitatively evaluating the organoid’s molecular profile relative to human tissues. However, the development of standardized differentiation protocols, spatial transcriptomics techniques, and multi-omic reference atlases has begun to address these limitations (Noack et al. 2023; Zenk et al. 2024).

One limitation is limited physiological complexity. Organoid cultures do not yet fully recapitulate the physiological complexity of the cell types of the developing brain, and, likewise, the absence of a functional vascular network limits the diffusion of oxygen and nutrients, resulting in frequent necrotic cores in cultured organoids (Quadrato et al. 2017; Tanaka et al. 2020; Cheruku et al. 2024; Konwarh 2025). The partial maturation of organoids complicates the modeling of neurodegenerative diseases, since the timing of disease emergence and symptoms depends on cellular aging, protein aggregation, and neural circuitry processes that occur only during aging in vivo and take many months to years to develop. An illustration of this dilemma is the case of organoids derived from familial AD cells, in which organoids that produce amyloid deposits or phosphorylated tau exhibit pathological phenotypes resembling those of an immature human cortex (Gonzalez et al. 2018).

Another limitation is reproducibility. Despite ongoing methodological development and refinement in culturing organoids, variability in size, morphological type, and cell types within a batch remains a major challenge. These problems are exacerbated in self-organizing or unguided methods (Quadrato et al. 2017; Tanaka et al. 2020).

Collectively, researchers have employed exogenous patterning approaches, micro-scaffolds to provide orientation, or even mini-bioreactors to improve organoid standardization (Kadoshima et al. 2013; Paşca et al. 2015; Qian et al. 2016; Pollen et al. 2019). Indeed, these approaches can help reduce variability. Still, human organoids will always have some inherent variability, which is particularly challenging when modeling disease (e.g., patient-derived lines to test a phenotype such as microcephaly (Lancaster et al. 2013) or autism disorders, and further distinguishing accurate pathophysiological response from background biological variability (Lancaster and Knoblich 2014; Mariani et al. 2015; Birey et al. 2017).

While interspecies comparisons are similarly constrained by biological considerations and may provide insight into both primate evolution and human-specific characteristics of cortical expansion, it can be challenging to observe differences when juxtaposing humans with non-humans based on developmental exposure and timing. As such, the timing of the organoid systems must be developed to generalize their root trajectories relative to these intrinsic occurrence trajectories across the two species. For instance, human progenitors cycle and proliferate on slower timescales than macaque or mouse cells, but these lineages then mirror the population’s growth rhythms (Mora-Bermúdez et al. 2016; Otani et al. 2016). Therefore, while this could, on the one hand, be a valuable comparative model for observing the relative characteristics of a biological system or model, deviations should be considered, particularly regarding developmental trajectories, before making assumptions and drawing conclusions about biological events during gestation or in the development of non-human primates.

In addition to biological limitations, the ethical landscape surrounding increasingly sophisticated organoid systems is shifting as rapidly as technology itself advances. Organoids develop network connectivity, synchrony, oscillations, and cross-frequency coupling and functional connectivity, which one could argue not only represent biological self-organization; some would say that proximity to networks of lower-order cortical circuits would demand deliberation over where we set potential thresholds for sentience, nociceptive awareness, or even fundamental aspects of sensory processing (Quadrato et al. 2017; Trujillo et al. 2019).

As evidence of organoids continues to grow and an increasing number of human and non-human primate organoid studies remain far from proving the complex, integrated neurological architecture required for self-awareness or for utilitarian moral or ethical acceptance, the progressive layers of complexity in organoid systems continue to demand scrutiny.

In simple life processes, in vitro biological systems must be evaluated against concepts that were previously a domain of animal research (e.g., network responsiveness, activity-dependent plasticity, or communication between regions) to be meaningful. The challenge is amplified by the rapid expansion of organoid incorporation with external technologies such as gene editing, optogenetics, and high-throughput monitoring and recording, which offer investigators precision for identifying or ontogenetically manipulating data at the time of organoid acquisition (Watanabe et al. 2017; Fischer et al. 2019; Shiri et al. 2019).

While combining these approaches offers powerful avenues not only for understanding disease mechanisms or identifying therapeutics, but also for reshaping ethical discussions that intersect with the production, development, and potential enhancement of a human-derived neuronal system.

As research trends toward combining organoids with technology to identify cell-level mechanisms of disease or to develop therapeutics for neurological diseases, including neurodegenerative diseases, it is more important than ever to establish robust oversight. Ethical frameworks should be designed to be responsive to the origins of advanced pluripotent stem-cell models, but also to include other layers related to developmental maturation processes, and even to the complexity reflected in the network of experimental criteria for organoid activity.

Mechanisms for oversight should also be developed alongside other similar fields, for associations or for exemptions, to maintain responsiveness and responsibility for all embryos and advance appreciation of regenerative medicine practices, while also modeling neurodevelopmental or developmental disorders, and possibly using evolutionary inquiry. There is, therefore, considerable enthusiasm about the neuroscience of brain organoids and their usefulness in constrained spaces. In the face of biological, technical, and ethical limits, do not diminish their potential value; instead, they impose a responsibility to build these models accurately with restraint and caution, even as they expand our understanding and appreciation of human biology and development.

## Conclusion

In the last ten years, cerebral organoids have gradually transitioned from a proof-of-concept technology to a versatile experimental platform for interrogating the developmental principles of the human brain. What began as an effort to induce pluripotent stem cells to develop into simple three-dimensional neural tissues has now become a platform that recapitulates key features of many of the cellular milestones, architectural transitions, and maturation processes of early corticogenesis.

While these systems remain entirely in vitro, they now occupy a unique position at the interface of model systems and living neural tissue. One of the clearest lessons from recent work with organoids is that they are not static representations of the fetal brain—they are dynamic, biological tissues with developmental trajectories that can be modulated, stabilized, or perturbed through defined culture conditions.

Advancements in bioreactor design, oxygenation, and long-term support have enabled organoids to survive longer, making it possible to achieve transcriptomic and functional maturation that were previously unavailable. When cultivating human neuronal tissues, functional neuronal networks emerge, establishing excitatory and inhibitory synaptic connections.

These milestones indicate not just structural maturation, but also the early development of functional circuitry. However, the limitations of in vitro maturation are equally informative. Hypoxia-driven necrosis at the core of older organoids, constraints on nutrient diffusion, and the absence of vasculature impose natural developmental limits that any media supplementation cannot erase.

At this stage of development, in vivo transplantation has opened new doors. When grafted into the rat brain, for example, organoids become vascularized by the host, promoting neuronal survival and maturation and giving rise to a much greater diversity of astrocytes, oligodendrocytes, and microglia. These hybrid models offer a glimpse of what organoids may become when relieved of key in vitro constraints.

Arguably, most importantly, the ability to consider organoids as an example of the in vitro-in vivo boundary offers a unique opportunity to observe how human neurons function as components of a living circuit system.

Following transplantation, organoids develop axons that extend into the host tissue, receive presynaptic input, and engage in local sensory processing—thus demonstrating not only that neurons may survive in the brain but also that they may integrate into local neural circuits. These observations do not recreate the full cortical lamination, long-range tracts, or the synchronous activity characteristic of primate networks. Yet, they clearly illustrate that organoid-derived neurons are functional and capable of circuit-like activity.

At the same time, organoid studies have begun to reveal the cellular vulnerabilities underpinning human neurological disorders. From congenital neurodevelopmental syndromes to degenerative disorders, organoids provide an experimentally controlled human-specific system for isolating, perturbing, and assessing human-specific pathways that would not be feasible in controlled in vivo settings. Transcriptional mapping, profiling neuronal activity, and creating region-specific organoid fusions reveal how subtle alterations in progenitor behavior or synaptic development can lead to widespread structural and functional abnormalities.

Together, these developments signal that the field is at an inflection point. Organoids are no longer limited to simplified developmental models; they are becoming tools for assessing and exploring human neuronal development, plasticity, and disease mechanisms. Their limitations must be acknowledged—not least for ethical reasons—but their potential must not be overlooked. As culture systems continue to improve and transplantation efforts become increasingly refined, organoids will begin to serve as an essential link between the controllable precision of in vitro biology and the complex, emergent features of a living brain.

In the future, advances in vascularization, immune cell involvement, long-term development, standardized differentiation protocols, and computational validation are expected to enhance the reproducibility and translational significance of brain organoids. However, these systems should be considered human-specific models that bridge the divide between classical cell culture methods and animal experiments. The main benefit of such systems is that they enable mechanistic insights that cannot be readily obtained with conventional experimental models.

## Data Availability

No datasets were generated or analysed during the current study.

## References

[CR114] Schier, A. F. (2003). Nodal signaling in vertebrate development. Annual Review of Cell and Developmental Biology, 19,589–621 10.1146/annurev.cellbio.19.041603.094522 14570583

[CR1] Abu-Khalil A et al (2004) Wnt genes define distinct boundaries in the developing human brain: implications for human forebrain patterning. J Comp Neurol 474(2):276–288 15164427 10.1002/cne.20112

[CR2] Amaya E, Musci TJ, Kirschner MW (1991) Expression of a dominant negative mutant of the FGF receptor disrupts mesoderm formation in *Xenopus* embryos. Cell 66(2):257–270 1649700 10.1016/0092-8674(91)90616-7

[CR3] Bain G et al (1995) Embryonic stem cells express neuronal properties in vitro. Dev Biol 168(2):342–357 7729574 10.1006/dbio.1995.1085

[CR4] Bennett D et al (2006) Neuropathology of older persons without cognitive impairment from two community-based studies. Neurology 66(12):1837–1844 16801647 10.1212/01.wnl.0000219668.47116.e6

[CR5] Bi F-C et al (2021) Optimization of cerebral organoids: a more qualified model for Alzheimer’s disease research. Translational Neurodegeneration 10(1):27 34372927 10.1186/s40035-021-00252-3 PMC8349709

[CR6] Birey F et al (2017) Assembly of functionally integrated human forebrain spheroids. Nature 545(7652):54–59 28445465 10.1038/nature22330 PMC5805137

[CR7] Blumberg B et al (1997) An essential role for retinoid signaling in anteroposterior neural patterning. Development 124(2):373–379 9053313 10.1242/dev.124.2.373

[CR8] Brickman JM, Serup P (2017) Properties of embryoid bodies. Wiley Interdiscip Rev Dev Biol 6(2):e259 10.1002/wdev.259 27911036

[CR9] Bystron I, Blakemore C, Rakic P (2008) Development of the human cerebral cortex: Boulder Committee revisited. Nat Rev Neurosci 9(2):110–122 18209730 10.1038/nrn2252

[CR10] Cakir B et al (2019) Engineering of human brain organoids with a functional vascular-like system. Nat Methods 16(11):1169–1175 31591580 10.1038/s41592-019-0586-5 PMC6918722

[CR11] Camps J et al (2025) Artificial intelligence-driven integration of multi-omics and radiomics: a new hope for precision cancer diagnosis and prognosis. Biochim Biophys Acta Mol Basis Dis 1871(6):167841 40216368 10.1016/j.bbadis.2025.167841

[CR12] Cataldo AM et al (2000) Endocytic pathway abnormalities precede amyloid β deposition in sporadic Alzheimer’s disease and Down syndrome: differential effects of APOE genotype and presenilin mutations. Am J Pathol 157(1):277–286 10880397 10.1016/s0002-9440(10)64538-5 PMC1850219

[CR13] Cataldo A et al (2001) Endocytic disturbances distinguish among subtypes of Alzheimer’s disease and related disorders. Annals Neurology: Official J Am Neurol Association Child Neurol Soc 50(5):661–665 11706973

[CR14] Chen V et al (2024) Applying interpretable machine learning in computational biology—pitfalls, recommendations and opportunities for new developments. Nat Methods 21(8):1454–1461 39122941 10.1038/s41592-024-02359-7 PMC11348280

[CR15] Chen M-M et al (2025) Construction Strategies and Challenges of Vascularized Brain Organoids. Progress Biochem Biophys 52(7):1757–1770

[CR16] Chenn A, Walsh CA (2002) Regulation of cerebral cortical size by control of cell cycle exit in neural precursors. Science 297(5580):365–369 12130776 10.1126/science.1074192

[CR17] Cheruku GR et al (2024) Recent advances and future perspectives in vascular organoids and vessel-on-chip. Organoids 3(3):203–246

[CR18] Chiaradia I, Lancaster MA (2020) Brain organoids for the study of human neurobiology at the interface of in vitro and in vivo. Nat Neurosci 23(12):1496–1508 33139941 10.1038/s41593-020-00730-3

[CR19] Choi SH et al (2014) A three-dimensional human neural cell culture model of Alzheimer’s disease. Nature 515(7526):274–278 25307057 10.1038/nature13800 PMC4366007

[CR20] Crossley PH, Martinez S, Martin GR (1996) Midbrain development induced by FGF8 in the chick embryo. Nature 380(6569):66–68 8598907 10.1038/380066a0

[CR21] Cuomo AS, HipSci Consortium et al (2020) Single-cell RNA-sequencing of differentiating iPS cells reveals dynamic genetic effects on gene expression. Nat Commun 11(1):810 10.1038/s41467-020-14457-z PMC7010688 32041960

[CR22] Doody RS et al (2013) A phase 3 trial of semagacestat for treatment of Alzheimer’s disease. N Engl J Med 369(4):341–350 23883379 10.1056/NEJMoa1210951

[CR23] Doody RS et al (2014) Phase 3 trials of solanezumab for mild-to-moderate Alzheimer’s disease. N Engl J Med 370(4):311–321 24450890 10.1056/NEJMoa1312889

[CR24] Eiraku M et al (2008) Self-organized formation of polarized cortical tissues from ESCs and its active manipulation by extrinsic signals. Cell Stem Cell 3(5):519–532 18983967 10.1016/j.stem.2008.09.002

[CR25] Eiraku M et al (2011) Self-organizing optic-cup morphogenesis in three-dimensional culture. Nature 472(7341):51–56 21475194 10.1038/nature09941

[CR26] Fiorenzano A et al (2021) Single-cell transcriptomics captures features of human midbrain development and dopamine neuron diversity in brain organoids. Nat Commun 12(1):7302 34911939 10.1038/s41467-021-27464-5 PMC8674361

[CR27] Fischer J, Heide M, Huttner WB (2019) Genetic modification of brain organoids. Front Cell Neurosci 13:558 31920558 10.3389/fncel.2019.00558 PMC6928125

[CR28] Fukuchi-Shimogori T, Grove EA (2001) Neocortex patterning by the secreted signaling molecule FGF8. Science 294(5544):1071–1074 11567107 10.1126/science.1064252

[CR29] Giandomenico SL, Sutcliffe M, Lancaster MA (2021) Generation and long-term culture of advanced cerebral organoids for studying later stages of neural development. Nat Protoc 16(2):579–602 33328611 10.1038/s41596-020-00433-w PMC7611064

[CR30] Gonzalez C et al (2018) Modeling amyloid beta and tau pathology in human cerebral organoids. Mol Psychiatry 23(12):2363–2374 30171212 10.1038/s41380-018-0229-8 PMC6594704

[CR31] Gordon A et al (2021) Long-term maturation of human cortical organoids matches key early postnatal transitions. Nat Neurosci 24(3):331–342 33619405 10.1038/s41593-021-00802-y PMC8109149

[CR32] Grass T et al (2024) Isogenic patient-derived organoids reveal early neurodevelopmental defects in spinal muscular atrophy initiation. Cell Rep Med. 10.1016/j.xcrm.2024.101659 39067446 PMC11384962

[CR33] Hansen DV et al (2010) Neurogenic radial glia in the outer subventricular zone of human neocortex. Nature 464(7288):554–561 20154730 10.1038/nature08845

[CR34] He C et al (2023) BOMA, a machine-learning framework for comparative gene expression analysis across brains and organoids. Cell Rep Methods. 10.1016/j.crmeth.2023.100409 36936070 PMC10014309

[CR111] Lui, J. H., Hansen, D. V., & Kriegstein, A. R. (2011). Development and evolution of the human neocortex. Cell, 146(1),18–36 10.1016/j.cell.2011.06.030 PMC3610574 21729779

[CR35] Jaworski T et al (2011) GSK-3α/β kinases and amyloid production in vivo. Nature 480(7376):E4–E5 22158250 10.1038/nature10615

[CR36] Jo J et al (2016) Midbrain-like organoids from human pluripotent stem cells contain functional dopaminergic and neuromelanin-producing neurons. Cell Stem Cell 19(2):248–257 27476966 10.1016/j.stem.2016.07.005 PMC5510242

[CR37] Johnstone M et al (2019) Reversal of proliferation deficits caused by chromosome 16p13. 11 microduplication through targeting NFκB signaling: an integrated study of patient-derived neuronal precursor cells, cerebral organoids and in vivo brain imaging. Mol Psychiatry 24(2):294–311 30401811 10.1038/s41380-018-0292-1 PMC6344377

[CR38] Jourdon A et al (2023) Modeling idiopathic autism in forebrain organoids reveals an imbalance of excitatory cortical neuron subtypes during early neurogenesis. Nat Neurosci 26(9):1505–1515 37563294 10.1038/s41593-023-01399-0 PMC10573709

[CR115] Chiang, C., Litingtung, Y., Lee, E., Young, K. E., Corden, J. L., Westphal, H., &Beachy, P. A. (1996). Cyclopia and defective axial patterning in mice lacking Sonic hedgehog gene function. Nature, 383(6599), 407–413 10.1038/383407a0 8837770

[CR39] Kadoshima T et al (2013) Self-organization of axial polarity, inside-out layer pattern, and species-specific progenitor dynamics in human ES cell–derived neocortex. Proc Natl Acad Sci 110(50):20284–20289 24277810 10.1073/pnas.1315710110 PMC3864329

[CR40] Kajtez J et al (2025) Three-dimensional cell-cell interactions promote direct reprogramming of patient fibroblasts into functional and transplantable neurons. Sci Adv 11(23):eadq7855 40479059 10.1126/sciadv.adq7855 PMC12143395

[CR41] Kawada J et al (2017) Generation of a motor nerve organoid with human stem cell-derived neurons. Stem cell Rep 9(5):1441–1449 10.1016/j.stemcr.2017.09.021 PMC5831012 29107592

[CR42] Kim S et al (2019) Generation, transcriptome profiling, and functional validation of cone-rich human retinal organoids. Proc Natl Acad Sci 116(22):10824–10833 31072937 10.1073/pnas.1901572116 PMC6561190

[CR43] Kim J et al (2025) Human assembloid model of the ascending neural sensory pathway. Nature 642(8066):143–153 40205039 10.1038/s41586-025-08808-3 PMC12137141

[CR44] Konwarh R (2025) Vascularized organoid-on-chip platforms: Expanding the bioengineering frontiers with microfluidics. Biomaterials Connect 2(1):1–10

[CR45] Kovacs GG et al (2013) Non-Alzheimer neurodegenerative pathologies and their combinations are more frequent than commonly believed in the elderly brain: a community-based autopsy series. Acta Neuropathol 126(3):365–384 23900711 10.1007/s00401-013-1157-y

[CR46] Kovacs GG, Lee VM, Trojanowski JQ (2017) Protein astrogliopathies in human neurodegenerative diseases and aging. Brain Pathol 27(5):675–690 28805003 10.1111/bpa.12536 PMC5578412

[CR47] Kriegstein A, Alvarez-Buylla A (2009) The glial nature of embryonic and adult neural stem cells. Annu Rev Neurosci 32(1):149–184 19555289 10.1146/annurev.neuro.051508.135600 PMC3086722

[CR113] Zhou, X., Sasaki, H., Lowe, L.,Hogan, B. L. M., & Kuehn, M. R. (1993). Nodal is a novel TGF-β-like gene expressed in the mouse node during gastrulation. Nature, 361(6412), 543–547 10.1038/361543a0 8429908

[CR48] Lancaster MA, Knoblich JA (2014) Generation of cerebral organoids from human pluripotent stem cells. Nat Protoc 9(10):2329–2340 25188634 10.1038/nprot.2014.158 PMC4160653

[CR49] Lancaster MA et al (2013) Cerebral organoids model human brain development and microcephaly. Nature 501(7467):373–379 23995685 10.1038/nature12517 PMC3817409

[CR50] Lancaster MA et al (2017) Guided self-organization and cortical plate formation in human brain organoids. Nat Biotechnol 35(7):659–666 28562594 10.1038/nbt.3906 PMC5824977

[CR51] Lee H-K et al (2016) Three dimensional human neuro-spheroid model of Alzheimer’s disease based on differentiated induced pluripotent stem cells. PLoS One 11(9):e0163072 27684569 10.1371/journal.pone.0163072 PMC5042502

[CR52] Li T et al (2025) Organoids and Organoids-on‐Chip in Traditional Chinese Medicine Research: Applications, Advantages, and Future Prospects. Cell Biol Int 49(10):1233–1244 40772545 10.1002/cbin.70067

[CR53] Liem KF et al (1995) Dorsal differentiation of neural plate cells induced by BMP-mediated signals from epidermal ectoderm. Cell 82(6):969–979 7553857 10.1016/0092-8674(95)90276-7

[CR54] Lin Y-T et al (2018) APOE4 causes widespread molecular and cellular alterations associated with Alzheimer’s disease phenotypes in human iPSC-derived brain cell types. Neuron 98(6):1141–1154 29861287 10.1016/j.neuron.2018.05.008 PMC6023751

[CR55] Lindenhofer D et al (2024) Cerebral organoids display dynamic clonal growth and tunable tissue replenishment. Nat Cell Biol 26(5):710–718 38714853 10.1038/s41556-024-01412-z PMC11098754

[CR56] Liu K et al (2025) From organoids to organoids-on-a-chip: Current applications and challenges in biomedical research. Chin Med J 138(07):792–807 39994843 10.1097/CM9.0000000000003535 PMC11970821

[CR57] Long KR, Huttner WB (2019) How the extracellular matrix shapes neural development. Royal Soc Open Biology 9(1):180216 10.1098/rsob.180216 PMC6367132 30958121

[CR58] Lu J et al (2021) Characterization of an in vitro 3D intestinal organoid model by using massive RNAseq-based transcriptome profiling. Sci Rep 11(1):16668 34404908 10.1038/s41598-021-96321-8 PMC8371140

[CR59] Luo C et al (2016) Cerebral organoids recapitulate epigenomic signatures of the human fetal brain. Cell Rep 17(12):3369–3384 28009303 10.1016/j.celrep.2016.12.001 PMC5495578

[CR60] Mansour AA et al (2018) An in vivo model of functional and vascularized human brain organoids. Nat Biotechnol 36(5):432–441 29658944 10.1038/nbt.4127 PMC6331203

[CR61] Mariani J et al (2015) FOXG1-dependent dysregulation of GABA/glutamate neuron differentiation in autism spectrum disorders. Cell 162(2):375–390 26186191 10.1016/j.cell.2015.06.034 PMC4519016

[CR62] McMahon AP, Bradley A (1990) The Wnt-1 (int-1) proto-oncogene is required for development of a large region of the mouse brain. Cell 62(6):1073–1085 2205396 10.1016/0092-8674(90)90385-r

[CR63] Miura Y et al (2022) Engineering brain assembloids to interrogate human neural circuits. Nat Protoc 17(1):15–35 34992269 10.1038/s41596-021-00632-z

[CR64] Mora-Bermúdez F et al (2016) Differences and similarities between human and chimpanzee neural progenitors during cerebral cortex development. Elife 5:e18683 27669147 10.7554/eLife.18683 PMC5110243

[CR65] Mukhopadhyay M et al (2001) Dickkopf1 is required for embryonic head induction and limb morphogenesis in the mouse. Dev Cell 1(3):423–434 11702953 10.1016/s1534-5807(01)00041-7

[CR66] Noack F et al (2023) Joint epigenome profiling reveals cell-type-specific gene regulatory programmes in human cortical organoids. Nat Cell Biol 25(12):1873–1883 37996647 10.1038/s41556-023-01296-5 PMC10709149

[CR67] Otani T et al (2016) 2D and 3D stem cell models of primate cortical development identify species-specific differences in progenitor behavior contributing to brain size. Cell Stem Cell 18(4):467–480 27049876 10.1016/j.stem.2016.03.003 PMC4826446

[CR68] Pankratz MT et al (2007) Directed neural differentiation of human embryonic stem cells via an obligated primitive anterior stage. Stem Cells 25(6):1511–1520 17332508 10.1634/stemcells.2006-0707 PMC2743478

[CR69] Park J et al (2018) A 3D human triculture system modeling neurodegeneration and neuroinflammation in Alzheimer’s disease. Nat Neurosci 21(7):941–951 29950669 10.1038/s41593-018-0175-4 PMC6800152

[CR70] Paşca AM et al (2015) Functional cortical neurons and astrocytes from human pluripotent stem cells in 3D culture. Nat Methods 12(7):671–678 26005811 10.1038/nmeth.3415 PMC4489980

[CR71] Phiel CJ et al (2003) GSK-3α regulates production of Alzheimer’s disease amyloid-β peptides. Nature 423(6938):435–439 12761548 10.1038/nature01640

[CR72] Pollen AA et al (2019) Establishing cerebral organoids as models of human-specific brain evolution. Cell 176(4):743–756 30735633 10.1016/j.cell.2019.01.017 PMC6544371

[CR73] Qian X et al (2016) Brain-region-specific organoids using mini-bioreactors for modeling ZIKV exposure. Cell 165(5):1238–1254 27118425 10.1016/j.cell.2016.04.032 PMC4900885

[CR74] Qu B et al (2024) Interpretation of the past, present, and future of organoid technology: an updated bibliometric analysis from 2009 to 2024. Front Cell Dev Biol 12:1433111 39193361 10.3389/fcell.2024.1433111 PMC11347291

[CR75] Quadrato G et al (2017) Cell diversity and network dynamics in photosensitive human brain organoids. Nature 545(7652):48–53 28445462 10.1038/nature22047 PMC5659341

[CR76] Raja WK et al (2016) Self-organizing 3D human neural tissue derived from induced pluripotent stem cells recapitulate Alzheimer’s disease phenotypes. PLoS One 11(9):e0161969 27622770 10.1371/journal.pone.0161969 PMC5021368

[CR77] Rakic P (1972) Mode of cell migration to the superficial layers of fetal monkey neocortex. J Comp Neurol 145(1):61–83 4624784 10.1002/cne.901450105

[CR110] Rakic, P. (2009). Evolution of the neocortex: A perspective from developmental biology. Nature Reviews Neuroscience, 10(10), 724–735 10.1038/nrn2719 PMC2913577 19763105

[CR78] Reumann D et al (2023) In vitro modeling of the human dopaminergic system using spatially arranged ventral midbrain–striatum–cortex assembloids. Nat Methods 20(12):2034–2047 38052989 10.1038/s41592-023-02080-x PMC10703680

[CR79] Rubenstein J, Rakic P (2013) Patterning and cell type specification in the developing CNS and PNS: comprehensive developmental neuroscience. Academic Press

[CR80] Sakaguchi H et al (2015) Generation of functional hippocampal neurons from self-organizing human embryonic stem cell-derived dorsomedial telencephalic tissue. Nat Commun 6(1):8896 26573335 10.1038/ncomms9896 PMC4660208

[CR81] Salloway S et al (2014) Two phase 3 trials of bapineuzumab in mild-to-moderate Alzheimer’s disease. N Engl J Med 370(4):322–333 24450891 10.1056/NEJMoa1304839 PMC4159618

[CR82] Schimelman J et al (2026) Biofabrication of Vascularized Tissues. Chem Rev 126(10):5852–5876 42127207 10.1021/acs.chemrev.5c00914

[CR83] Seo J et al (2017) Inhibition of p25/Cdk5 attenuates tauopathy in mouse and iPSC models of frontotemporal dementia. J Neurosci 37(41):9917–9924 28912154 10.1523/JNEUROSCI.0621-17.2017 PMC5637118

[CR84] Shah N (2024) Multi-Software Comparison for Calcium Flux Analysis of iPSC. -Derived Cardiac Tissue.

[CR85] Shen Y-P, Kokaia Z (2025) Brain organoid transplantation: a comprehensive guide to the latest advances and practical applications—a systematic review. Cells 14(14):1074 40710327 10.3390/cells14141074 PMC12293213

[CR86] Shiri Z et al (2019) Optogenetics in the era of cerebral organoids. Trends Biotechnol 37(12):1282–1294 31227305 10.1016/j.tibtech.2019.05.009

[CR87] Simintiras CA et al (2021) Capture and metabolomic analysis of the human endometrial epithelial organoid secretome. Proc Natl Acad Sci U S A 118(15):e2026804118 33876774 10.1073/pnas.2026804118 PMC8053979

[CR88] Smits LM et al (2019) Modeling Parkinson’s disease in midbrain-like organoids. NPJ Parkinson’s disease 5(1):5 30963107 10.1038/s41531-019-0078-4 PMC6450999

[CR89] Song Y et al (2026) Vascularised brain organoids: engineering strategies and neurobiological applications. Cell Prolif 59(3):e70161 41521070 10.1111/cpr.70161 PMC12961551

[CR90] Stachowiak E et al (2017) Cerebral organoids reveal early cortical maldevelopment in schizophrenia—computational anatomy and genomics, role of FGFR1. Translational psychiatry 7(11):6 30446636 10.1038/s41398-017-0054-x PMC5802550

[CR91] Subramanian L et al (2009) Signals from the edges: the cortical hem and antihem in telencephalic development. *Seminars in cell* & *developmental biology*, Elsevier, pp. 712–718 10.1016/j.semcdb.2009.04.001 PMC2791850 19446478

[CR92] Takebe T et al (2015) Vascularized and complex organ buds from diverse tissues via mesenchymal cell-driven condensation. Cell Stem Cell 16(5):556–565 25891906 10.1016/j.stem.2015.03.004

[CR93] Tanaka Y et al (2020) Synthetic analyses of single-cell transcriptomes from multiple brain organoids and fetal brain. Cell Rep 30(6):1682–1689 32049002 10.1016/j.celrep.2020.01.038 PMC7043376

[CR94] Trapnell C (2024) Revealing gene function with statistical inference at single-cell resolution. Nat Rev Genet 25(9):623–638 38951690 10.1038/s41576-024-00750-w

[CR95] Trujillo CA et al (2019) Complex oscillatory waves emerging from cortical organoids model early human brain network development. Cell Stem Cell 25(4):558–569 31474560 10.1016/j.stem.2019.08.002 PMC6778040

[CR96] Ueda HR et al (2020) Tissue clearing and its applications in neuroscience (vol 21, pg 61, 2020), *NATURE REVIEWS NEUROSCIENCE* [Preprint] 10.1038/s41583-020-0291-5 32152524

[CR97] Van Duinen V et al (2015) Microfluidic 3D cell culture: from tools to tissue models. Curr Opin Biotechnol 35:118–126 26094109 10.1016/j.copbio.2015.05.002

[CR98] Velasco S et al (2019) Individual brain organoids reproducibly form cell diversity of the human cerebral cortex. Nature 570(7762):523–527 31168097 10.1038/s41586-019-1289-x PMC6906116

[CR99] Wang Q et al (2021) The dynamics of metabolic characterization in iPSC-derived kidney organoid differentiation via a comparative omics approach. Front Genet 12:632810 33643392 10.3389/fgene.2021.632810 PMC7902935

[CR100] Watanabe K et al (2005) Directed differentiation of telencephalic precursors from embryonic stem cells. Nat Neurosci 8(3):288–296 15696161 10.1038/nn1402

[CR101] Watanabe M et al (2017) Self-organized cerebral organoids with human-specific features predict effective drugs to combat Zika virus infection. Cell Rep 21(2):517–532 29020636 10.1016/j.celrep.2017.09.047 PMC5637483

[CR102] Wilson PA, Hemmati-Brivanlou A (1995) Induction of epidermis and inhibition of neural fate by Bmp-4. Nature 376(6538):331–333 7630398 10.1038/376331a0

[CR103] Wimmer RA et al (2019) Human blood vessel organoids as a model of diabetic vasculopathy. Nature 565(7740):505–510 30651639 10.1038/s41586-018-0858-8 PMC7116578

[CR104] Xekardaki A et al (2014) Neuropathological changes in aging brain, *Genedis 2014: Geriatrics*, pp. 11–17 10.1007/978-3-319-08939-3_6 25416106

[CR105] Xiang Y et al (2017) Fusion of regionally specified hPSC-derived organoids models human brain development and interneuron migration. Cell Stem Cell 21(3):383–398 28757360 10.1016/j.stem.2017.07.007 PMC5720381

[CR106] Zenk F et al (2024) Single-cell epigenomic reconstruction of developmental trajectories from pluripotency in human neural organoid systems. Nat Neurosci 27(7):1376–1386 38914828 10.1038/s41593-024-01652-0 PMC11239525

[CR112] Zhao, Y., Landau, S., Okhovatian, S., Liu, C., Lu, R. X., Lai, B. F. L., Wu, Q., Kieda, J., Cheung, K.,Rajasekar, S., Jozani, K., Zhang, B., & Radisic, M. (2024). Integrating organoids and organ-on-a-chip devices. Nature Reviews Bioengineering, 2, 588–608 10.1038/s44222-024-00207-z

[CR107] Zhang S-C et al (2001) In vitro differentiation of transplantable neural precursors from human embryonic stem cells. Nat Biotechnol 19(12):1129–1133 11731781 10.1038/nbt1201-1129

[CR108] Zhang H et al (2025) Brain organoids-on-chip for neural diseases modeling: history, challenges and trends. J Pharm Anal. 10.1016/j.jpha.2025.101323 41208919 PMC12594919

